# Pangenome-scale annotation of mycobacteriophages for dissecting phage–host interactions based on a sequence clustering and structural homology analysis strategy

**DOI:** 10.1128/msystems.00508-25

**Published:** 2025-07-29

**Authors:** Xiao Guo, Zheng-Guo He

**Affiliations:** 1 College of Life Science and Technology, Guangxi University 622308 , Nanning, China; 2 State Key Laboratory of Virology and Biosafety, Taikang Center for Life and Medical Sciences, TaiKang Medical School (School of Basic Medical Sciences), Wuhan University, Wuhan, China; Politecnico di Torino, Turin, Piemonte, Italy

**Keywords:** *Mycobacterium tuberculosis*, phage, structural homology, sequence clustering

## Abstract

With the increasing severity of bacterial drug resistance, there is a growing need for phages with well-defined genetic backgrounds to combat drug-resistant infections. Mycobacteriophages constitute the largest genome-sequenced phage group; however, the vast majority of these phage proteins have not yet been effectively annotated. In this study, we employed a structure-based similarity search approach to improve protein annotation. Through the application of this approach to 240,754 proteins from 2,169 mycobacteriophage genomes, we increased the protein annotation rate from 34% to 52.11%. Additionally, we identified a series of predicted counter-defense proteins, including anti-CRISPR proteins and antitoxins, and inferred the potential interaction network of phage-encoded proteins involved in replication, transcription, and translation with host-associated molecular machinery. This study addresses a substantial gap in the current knowledge of the potential function of phage proteins and provides key insights into the interactions between mycobacteriophages and their hosts.

**IMPORTANCE:**

Mycobacteriophages constitute the largest group of phages with sequenced genomes. However, a significant portion of these phage proteins have not yet been effectively annotated, seriously hindering our understanding of the basic biological processes of phage–host interactions and their practical applications. This study utilized a structure-based similarity search approach to enhance phage protein annotation. This approach led to the identification of novel predicted protein folds, structural domain fusion phenomena, and putative new enzymes. Additionally, the study identified a series of phage-encoded proteins that may play a role in hijacking host-associated replication, transcription, and translation processes, providing insights into the molecular mechanisms underlying mycobacteriophage interactions with host machinery. This study addresses a critical knowledge gap regarding the potential function of phage proteins and provides key insights into the interactions between mycobacteriophages and their hosts.

## INTRODUCTION

With the increase in drug-resistant bacteria and the delay in antibiotic development, antimicrobial resistance poses a threat to global public health and development (1). Consequently, alternative antibiotic therapies are being explored (2). Phages, a diverse group of bacterial viruses, have been investigated as potential agents for targeting drug-resistant bacterial pathogens (3).

In recent years, advancements in sequencing technology, particularly the emergence of next-generation sequencing methods, have significantly increased phage genomic data (4). The application of high-throughput metagenomics in studying the gut and global viromes has enlarged phage diversity (5, 6). Further investigation of phage genomic resources is supported by ongoing searches for phages within the existing microbiomes (7). Moreover, various initiatives for phage discovery, such as the Science Education Alliance-Phage Hunters Advancing Genomics and Evolutionary Science program, aim to expand the phage communities associated with specific hosts (8). However, the functions of numerous phage proteins remain unknown (9, 10).

Protein structural similarity-based homolog search offers greater sensitivity compared to sequence similarity-based search processes. Current methods for annotating phage proteins primarily depend on DNA or protein homology. However, phages frequently encode proteins without known homologs, posing a significant challenge in identifying distant evolutionary relationships through sequencing alone (10). Structural homology searches, which are 4–10 times more conservative than sequence-based searches, offer significant advantages for identifying distantly related protein homologs (11).

Annotations based on structural similarities are currently employed for annotating eukaryotic viromes and phage genomes. Within eukaryotic viromes, 38% of proteins have been identified using structural similarity searches in the AlphaFold database (12). For phage genomes, employing structural similarity searches has increased the identification rate of protein homologs from 10% to 40% in contrast to sequence homology searches (13). However, the substantial computational expense of structural prediction poses significant challenges for annotating phage proteins on a large scale.

Mycobacteriophages represent the largest and most extensively studied group of phages. Various case studies have demonstrated their potential in treating drug-resistant mycobacteria (14). In 2019, mycobacteriophages were initially utilized in the treatment of human mycobacterial infections, leading to significant clinical improvements in patients suffering from drug-resistant *Mycobacterium abscessus* (15). Subsequently, in 2022, these phages were reapplied for treating drug-resistant *M. abscessus* infections. Following successful phage therapy, a patient underwent lung transplantation, and no traces of *M. abscessus* were detected in the explanted lungs (16). During the same year, mycobacteriophage therapy was extended to treat drug-resistant *Mycobacterium chelonae* infections, resulting in significant amelioration of skin lesions in patients treated with a single bacteriophage (Muddy) in combination with antimicrobial therapy (17). Additionally, mycobacteriophages have been observed to glycosylate their capsid and tail tube proteins using intrinsic glycosyltransferases. This process shields viral particles from antibody binding and diminishes the production of neutralizing antibodies (18). Currently, only 35% of mycobacterial phage genomes have been annotated. Enhancing our understanding of the functional roles of phage-encoded genes would greatly benefit the development of engineered phages for therapeutic and other applications (19).

The prolonged conflict between mycobacteriophages and their hosts has resulted in complex phage–host interactions. Mycobacteria possess various defense mechanisms, including restriction-modification (20), toxin-antitoxin (TA) (21), and CRISPR-Cas systems (22). Mycobacteriophages have also been reported to encode defenses against superinfections, such as gp29-gp30 (ppGpp-mediated system) (23), CarolAnn gp43-gp44 (membrane potential loss-mediated system) (24), and Splash gp30-31 (repressor-associated system) (25). Another aspect of phage–host interactions involves competition for molecular machines necessary for replication, transcription, and translation. Phage genomes are highly streamlined and do not encode the full set of molecular machinery required for replication, transcription, and translation. Instead, they encode specific elements that enable them to subvert and utilize the host’s molecular systems for their own replication. Various components encoded by replication, transcription, and translation have been identified in mycobacterial phages (26, 27), although their specific roles in competing for the host’s molecular machinery remain unclear.

In this study, we developed a sequence clustering and structural homology-based annotation workflow (SCSH strategy). By utilizing sequence clustering and a structural homology-based approach, we effectively annotated a large data set of mycobacteriophage proteins, increasing the proportion of annotated proteins from 34% to 52.11%. Structural homology analysis of mycobacteriophage proteins and their closest structural matches revealed potential evolutionary relationships between mycobacteriophages and diverse biological domains, showing significant similarities in eukaryotic proteins. This analysis also predicted the presence of previously uncharacterized protein folds, potential structural domain fusion events, and putative new enzymes. Integrating the structural annotation results with the protein–protein interaction prediction results enabled the identification of candidate phage-encoded anti-defense proteins and the proposed potential anti-defense mechanisms. Furthermore, our findings offer insights into how mycobacteriophages utilize host replication-related, transcription-related, and translation-related proteins to achieve efficient reproduction.

## RESULTS

### Structural re-annotation of mycobacteriophage proteins through SCSH strategy

To achieve a comprehensive structural similarity-based re-annotation of gene functions encoded by mycobacteriophages, we obtained 240,754 protein sequence information from 2,169 mycobacteriophages retrieved from GenBank (Table S1).

**Fig 1:**
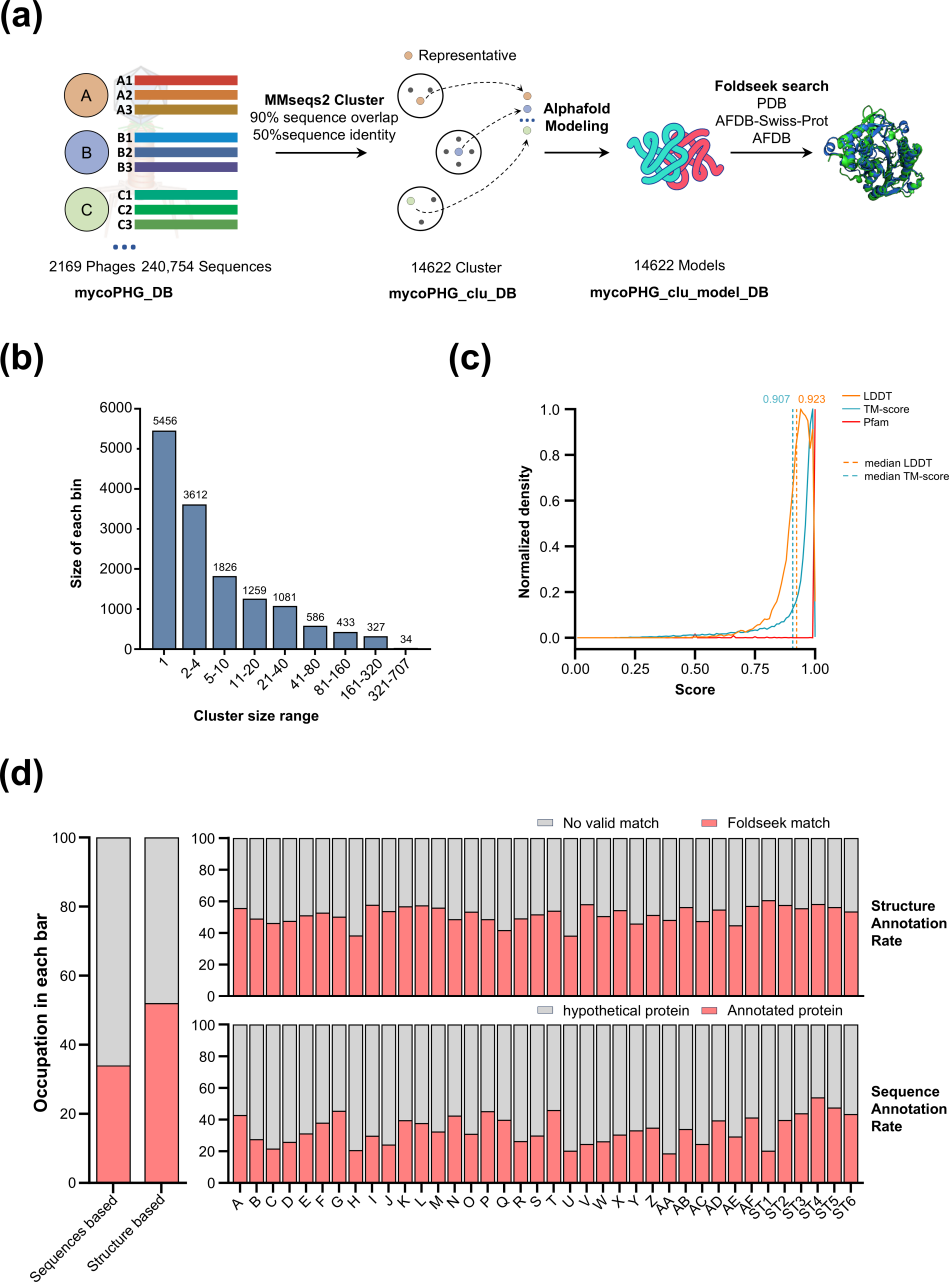
SCSH workflow and clusters summary. (**a**) A three-step SCSH workflow was used to annotate mycobacteriophages. First, MMseqs2 clustered 240,754 mycobacteriophage proteins with 50% sequence similarity and 90% sequence overlap, reducing the database to 14,622 clusters. Subsequently, AlphaFold predicted the structure of the representative sequence for each cluster. Finally, Foldseek was utilized to identify the optimal match for each representative structure in PDB, AFDB_SwissProt, and AFDB. (**b**) Summary of cluster distributions for different cluster sizes. (**c**) mycoPHG_clu_DB structural and Pfam consistency. The clusters exhibit a median LDDT of 0.923 and a median template modeling (TM) score of 0.907. Among all clusters, 94.8% with Pfam annotations exhibit 100% consistency. (**d**) Summary of sequence-based and Foldseek-based annotation rates across different phage clusters is presented in three panels. The left panel illustrates the average sequence annotation rate (34.00%) and the average structural annotation rate (52.11%) for 2,169 mycobacteriophages. The upper right panel displays the average structural-based annotation rate for each phage cluster, while the lower right panel shows the average sequences-based annotation rate for each phage cluster.

Our structural similarity-based annotation workflow comprised three primary steps (Fig. 1a): clustering, modeling, and structural similarity search. In the first step, the MMseqs2 (28) was utilized to cluster mycoPHG_DB at a 50% sequence identity and 90% sequence alignment overlap of both sequences (see Materials and Methods), resulting in 14,614 clusters. The size distribution of these clusters is shown in Fig. 1b. In the second step, AlphaFold (29) was employed to predict the structure of representative sequences within each cluster (see Materials and Methods). The model quality of each cluster was evaluated using the predicted local distance difference test (pLDDT). The median pLDDT was 77.06, and a positive correlation was observed between the cluster pLDDT and its size, as shown in Fig. S1b. Even singleton clusters displayed structures with high pLDDT values. Further analysis of the multiple sequence alignment (MSA) depth corresponding to different cluster sizes (Fig. S1c through e) revealed that 39.33% of protein MSAs in singleton clusters had a depth exceeding 100, with 27.69% exceeding 1,000 (Fig. S1c). This trend was more pronounced with the increasing cluster size, indicating that proteins in singleton clusters exhibited high pLDDT due to their relatively deep MSAs. In the third step, Foldseek (30) was employed to search for structural homologs of representative structures from mycoPHG_clu_DB (mycoPHG_clu_model_DB) across the protein data bank (PDB), AlphaFold protein structure database (AFDB)_SwissProt, and AFDB databases (see Materials and Methods). The contribution of each step to the annotation rate is shown in Fig. S1a. Annotation rates based on the annotation pipeline and sequence homology retrieved from GenBank are presented in Fig. 1d.

Taken together, utilizing the new SCSH strategy, the annotation rate of mycobacteriophage proteins increased from 34.0% to 52.11%.

### Cluster purity and annotation consistency analysis

Because our workflow operates by transferring structural annotations from cluster representatives to all proteins belonging to the same sequence-based cluster, it is crucial to assess the structural and functional consistency of these sequence-based clusters. Considering the limited availability of phage-related proteins in structural databases and the general lack of Pfam and Enzyme Commission (EC) annotations, we assessed the quality of our sequence-based clusters by evaluating the consistency of their structural and Pfam annotations (Fig. 1c) using the AFDB_SwissProt databases (13, 31).

By aligning each member with its representative, we observed that the clusters tended to be structurally homogeneous, as indicated by two structural similarity metrics (median LDDT of 0.923 and median template modeling [TM] score of 0.907; Fig. 1c). Similarly, members within the same cluster frequently shared identical Pfam domains (Fig. 1c), with 94.8% of cluster members displaying 100% consistency. The relationship between the number of cluster members and the consistency scores (Fig. S2a) demonstrates that clusters exhibit perfect consistency regardless of whether they include only a few or thousands of members. We further investigated the relationship between structural and functional similarities using Pfam (Fig. S2b) and EC annotations (Fig. S2c through f). Our clusters demonstrated high consistency across all LDDT levels. For EC (level 4), even within the lowest LDDT range (0.00, 0.65], 89.4% of clusters exhibit 100% of EC consistency, highlighting the robustness of our clustering approach.

We also assessed the consistency between structure-based annotations and existing sequence-based annotations. As expected, an increase in pLDDT score correlated with improved annotation consistency. Notably, with an increase in the pLDDT score from 0 to 88, the proportion of consistent annotations increased from 72.03% to 96.87% (Fig. S2g). Furthermore, we explored the relationship between cluster size and annotation consistency and found no significant correlation between these factors (Fig. S2h). These findings suggest that the primary determinant of the annotation accuracy is the confidence of the structure predicted by AlphaFold.

### Structural homology of mycobacteriophage proteins with eukaryotic homologs

**Fig 2:**
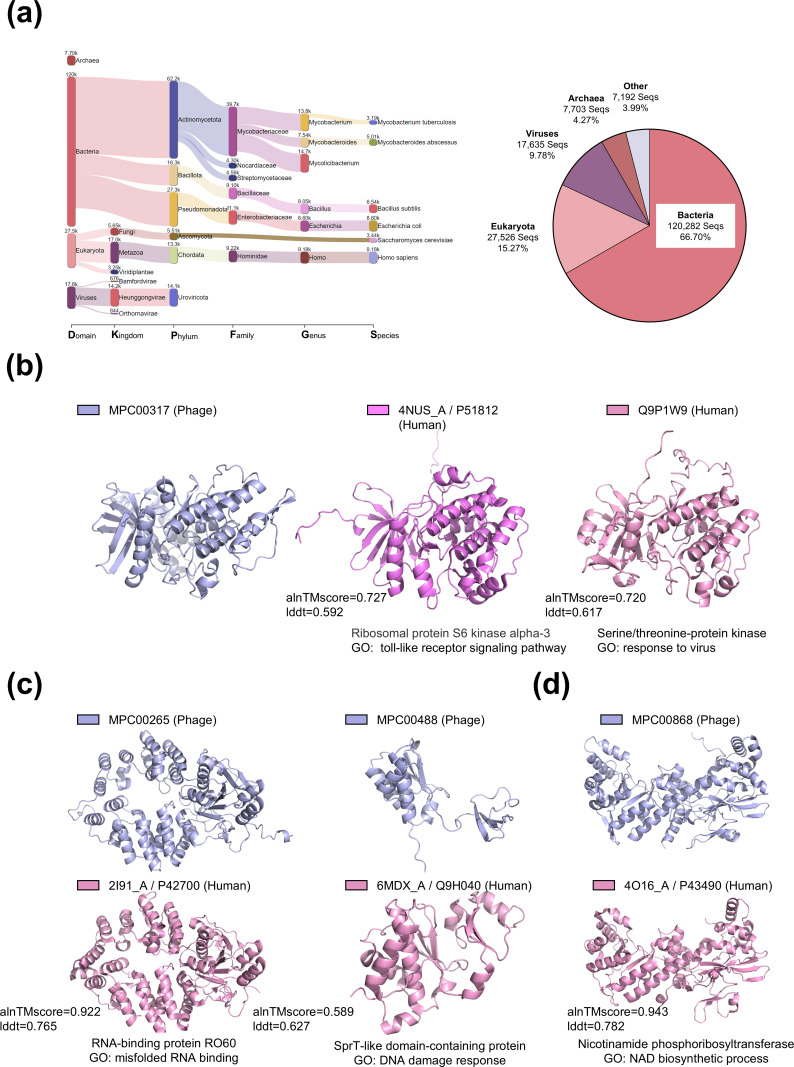
Homology between phage and eukaryote-encoded proteins. (**a**) Visualization of the last common ancestor of all structural homologies of phage proteins as a Sankey plot generated by Pavian (left chart). Only the largest eight taxonomical nodes per rank are shown. The percentage of each super-kingdom is depicted in the pie chart to the right panel. (**b**) The structural homolog of a phage protein (left) in *Homo sapiens* is depicted. The most suitable match for this protein in the PDB database is ribosomal protein S6 kinase alpha-3 (middle), whereas the top match in AFDB_SwissProt is serine/threonine-protein kinase (right). The human homologs of this phage protein are engaged in immune response-related pathways, demonstrating the cross-kingdom homology of immune-related proteins. (**c**) Two examples of phage proteins exhibit structural homology to proteins involved in fundamental life processes in human cells. The first example is an RNA-binding protein, RO60, which is annotated with a GO term for binding misfolded RNA, recognizing, binding, and potentially promoting the degradation of misfolded RNA. The second example is a protein with a SprT-like structural domain, annotated with a DNA damage response GO term, which repairs DNA-protein crosslinks to maintain genomic stability (right). (**d**) Examples of phage version nicotinamide phosphoribosyltransferase, annotated with a NAD biosynthetic process GO term. This enzyme catalyzes the synthesis of nicotinamide mononucleotide (NMN) from nicotinamide (NAM) and 5-phosphoribosyl-1-pyrophosphate (PRPP), representing a crucial rate-limiting step in the NAD salvage synthesis pathway.

To determine the taxonomic distribution of the best structural homology of mycobacteriophage hits, we analyzed their taxonomic compositions to identify homology across different domains (Fig. 2a). To investigate this further, the best hits were mapped to the Tree of Life, and the most recent common ancestor was identified for all hits (see Materials and Methods). Through this approach, we mapped the taxonomic distribution of the best hits across the bacteria (66.70%), eukaryota (15.27%), viruses (9.78%), and archaea (4.27%).

As anticipated, the taxonomic distribution of the majority of best hits corresponded to the primary host range of mycobacteriophages, including slow-growing mycobacterium and fast-growing mycolicibacterium. Interestingly, the eukaryotic domain ranked second among the mapped domains, with *Homo sapiens* and *Saccharomyces cerevisiae* being the most frequently mapped species (Fig. 2a). This suggests a potential functional connection between mycobacteriophages and eukaryotes.

### A eukaryotic-like phage-helper protein

Recent studies have shown that mycobacteriophage TM4 relies on a eukaryotic-like Ser/Thr protein kinase from the host to suppress and evade anti-phage immunity (32). Structural similarity analyses revealed that cluster C mycobacteriophages contain a protein with structural homology to eukaryotic-like Ser/Thr protein kinase (MPC00317). The most structurally analogous protein to MPC00317 in the PDB is ribosomal protein S6 kinase alpha-3 (PDB: 4NUS_A/UniProt: P51812) (33). Ribosomal protein S6 kinase alpha-3 is known to influence the ERK signaling pathway, which is crucial for HIV-1 replication and infection (34). By comparing the structure of MPC00317 to the AFDB_SwissProt database, the closest structural homolog to MPC00317 is identified as Ser/Thr protein kinase pim-2 (UniProt: Q9P1W9). The GO annotation of PIM2 indicated its involvement in the viral response (35) (Fig. 2b). The structural homologs of MPC00317 in the PDB and Swiss-Prot databases suggest its potential as a Ser/Thr protein kinase involved in viral infection. Structural alignment of MPC00317 with MSMEG_1200, 4NUS_A, and Q9P1W9, and calculation of the root-mean-square deviation (RMSD) showed that MPC00317 matched the StpK7 kinase domain with an RMSD of 1.557 (Fig. S3a and b).

This structural similarity indicates that the phage version Ser/Thr protein kinase might also contribute to suppressing the host immune response against the phage.

### The phage version of eukaryotic-like DNA/RNA-binding protein

Structural homology analysis indicated that some mycobacteriophages encode proteins that may exhibit structural similarities with proteins involved in fundamental cellular processes in human cells (Fig. 2c). For example, cluster C and AF mycobacteriophages (MPC00265) encode a protein that is structurally analogous to the human RNA-binding protein RO60 (PDB: 2I91_A/UniProt: P42700), which binds to misfolded non-coding RNAs and likely functions in RNA quality control (36). The SprT-like protein (MPC00488), which widely exists in mycobacteriophages, also exhibits structural homology with the human SprT-like domain protein (PDB: 6MDX_A/UniProt: Q9H040), which is crucial for maintaining genome integrity (37). The structural alignments and RMSD of MPC00265 with 2I91_A and MPC00488 with 6MDX_A are shown in Fig. S3c.

### Phage-encoded NAMPT may evade Thoeris defense

Additionally, cluster L phages encode a protein that has structural similarity to human nicotinamide phosphoribosyltransferase (NAMPT; NMPRTase, UniProt: P43490), the rate-limiting enzyme in the NAD salvage pathway from NAM (38) (Fig. 2d), with a nearly identical structural superposition (Fig. S3c). Interestingly, the currently reported NAD^+^ depletion-based phage defense system Thoeris includes the NAD^+^ hydrolyzing component ThsA, which catalyzes the hydrolytic cleavage of NAD to yield the product NAM (39). The structural similarity between NAMPT and the cluster L phage protein suggests the potential for counteracting abortive bacterial infections involving NAD^+^ depletion, although the precise functional role is yet to be determined.

### Structure-based clustering and functional homology across mycobacteriophage clusters

**Fig 3:**
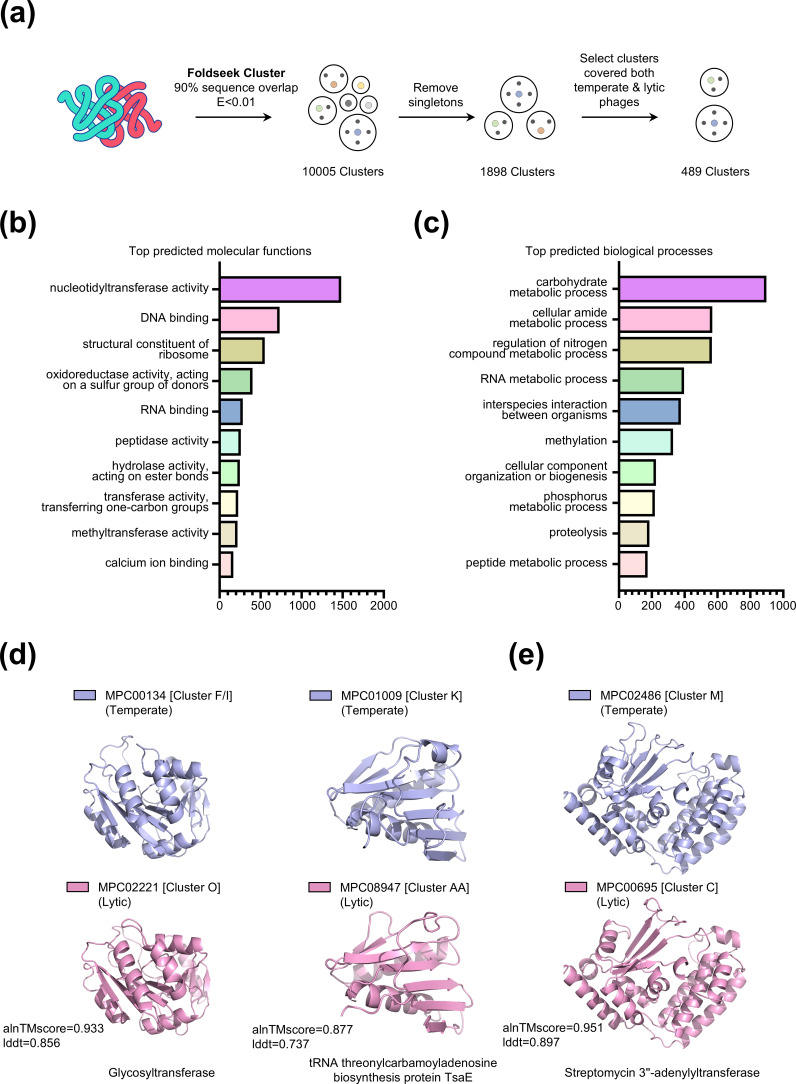
Workflow for structural clustering and functionality analysis of proteins shared between temperate and lytic phages. (**a**) A three-step structure cluster pipeline was utilized to discover functional homology among temperate and lytic mycobacteriophages. First, Foldseek was used to cluster 14,622 representative structures, applying a 90% sequence overlap criterion and an E-value of <0.01 for each structure alignment. Subsequently, clusters with a minimum of two structures were chosen. In the final step, the structural clusters were screened to detect those including both temperate phages and lytic phages within their members, resulting in 489 eligible structural clusters. (**b**) Counts of GO molecular function terms most frequently predicted by DeepFRI on the set of 826cluster representative structures with an average pLDDT score above 90. (**c**) Counts of GO biological processes terms most frequently predicted by DeepFRI on the set of 826 cluster representative structures with an average pLDDT score above 90. (**d**) Two examples of structurally homologous proteins found in temperate and lytic phages are discussed. The first instance is glycosyltransferase, which is encoded in cluster F and I temperate phages and cluster O virulent phages. Phages avoid detection by the human immune system through glycosylation of their proteins using glycosyltransferases. The second illustration is the tRNA threonylcarbamoyladenosine biosynthesis protein TsaE, present in K-cluster temperate phages and AA-cluster virulent phages. TsaE functions as a protein kinase with Ser/Thr/Tyr kinase and ATPase activities, potentially influencing protein translation regulation. (**e**) An example of a phage-encoded antibiotic resistance-associated protein is the streptomycin 3″-adenylyltransferase, which is encoded in M-cluster temperate phages and C-cluster virulent phages. This enzyme modifies streptomycin and spectinomycin, inhibiting their binding to ribosomal counterparts and conferring drug resistance.

Mycobacteriophages can be classified into different clusters with low average nucleotide identities (ANIs) between clusters (40). Structure-based homology searches can enhance sensitivity, enabling the identification of long-range protein homology across mycobacteriophage clusters. The Foldseek clustering module was employed to group the representative models of mycobacteriophage proteins (see Materials and Methods), yielding 10,005 clusters. After filtering out singleton clusters, 1,898 non-singleton clusters remained. Among these, 489 clusters, including both temperate and lytic phages, were chosen (Fig. 3a).

The structural consistency of the mycoPHG_clu_model_DB clusters (Fig. S4d through g) was assessed by aligning each cluster member with a representative structure. Our analysis revealed that the clusters tend to be structurally homogeneous, as indicated by two key structural similarity metrics: a median LDDT of 0.71 and a median TM score of 0.74 (Fig. S4d). Furthermore, the relationship between cluster size and these metrics was examined, revealing consistent TM-score and LDDT distributions across different cluster sizes, with no significant correlation with cluster size (Fig. S4e and f). Moreover, the TM-score distribution showed a positive association with the LDDT (Fig. S4g), further supporting the structural reliability of the clusters.

DeepFRI (41) was utilized to assign mycoPHG_clu_model_DB to the GO terms, and the EC number was predicted to identify novel enzymes (see Materials and Methods). For certain proteins where Foldseek could not identify valid matches, DeepFRI successfully annotated the GO terms and EC numbers, as shown in Fig. S1f. Furthermore, DeepFRI’s capability to annotate EC numbers in the mycobacteriophage data set is shown in Fig. S4h through j. To benchmark DeepFRI’s performance, we refined the set of DeepFRI-annotated phage proteins by selecting those whose best UniProt matches also contained EC annotations. This allowed for a direct comparison between the EC numbers predicted by DeepFRI and those documented in UniProt (see Materials and Methods). Based on this comparison, the agreement rates for the annotation of EC classes 1–3 were 74.77%, 65.42%, and 62.61%, respectively. To further assess the false positive rate of DeepFRI in the context of the mycobacteriophage data set, a validation set comprising 48 clusters–containing a total of 1,304 protein sequences–with high-confidence representative structural models (pLDDT > 90) was constructed. These clusters were annotated as structural proteins based on both structure-based and sequence-based annotation. Within this curated data set, 9.05% of proteins were predicted to have EC numbers by DeepFRI. Moreover, the proportion of high-confidence EC annotations (score > 0.8) was 3.14% (Table S15), highlighting their effectiveness in functional prediction.

To investigate the molecular functions and associated biological processes of the genes shared between the lytic and temperate phages, we applied a pLDDT threshold to minimize false positives, focusing solely on the DeepFRI annotation results corresponding to protein structures with a pLDDT score exceeding 90. The most frequently predicted molecular function and biological process results of the clusters encompassing both temperate and lytic phages are presented in Fig. 3b and c. The most commonly predicted molecular function was “nucleotidyltransferase activity” (1,482 proteins), and the most frequently predicted biological process was “‘carbohydrate metabolic process” (898 proteins).

### Widespread presence of glycosylation protein in mycobacteriophages

Mycobacteriophages can protect their virion particles from antibody binding by glycosylating their own proteins. Glycosylated proteins have been identified in the F-cluster mycobacteriophage Che8, O-cluster phage Corndog, and C-cluster phage Myrna (18). Cross-template-lytic structural-alignment-based clustering indicated that the two essential glycosyltransferases for viral capsid glycosylation, F-cluster phage Che8 gp110 and O-cluster phage Corndog gp38, were clustered together (Fig. 3d, SCM00150). The glycosyltransferase of C-cluster phage Myrna is classified within a lytic phage-specific structural cluster (Fig. S4a). This structural cluster also encompassed cluster I and singleton phages, implying that coat protein glycosylation might be a common strategy among various mycobacteriophages.

### Regulatory role of mycobacteriophage TsaE in host protein translation

Some mycobacteriophages encode proteins that exhibit structural similarity to TsaE, a protein linked to t^6^A (N6-threonylcarbamoyl adenosine) synthesis and are classified as novel Ser/Thr/Tyr kinases. Structural homology searches revealed phage proteins resembling TsaE, found in cluster K and cluster AA phages (Fig. 3d; Fig. S4b). TsaE plays a role in protein translation and oxidative stress responses in *Bacillus subtilis* (42). Owing to the limited number of phage proteins identified in large-scale interaction analyses that interact with the host translation system (Fig. 6a and b), phage-encoded variants of TsaE-like proteins may also influence protein translation regulation.

### Potential role of mycobacteriophages in host antibiotic resistance

Some mycobacteriophages encode proteins with structural similarities to aminoglycoside nucleotidyltransferases, which have been reported to mediate nucleotide modifications of streptomycin and spectinomycin. These modifications prevent drugs from binding to their corresponding sites on the ribosome, thereby conferring a drug-resistant phenotype to the bacterial host (43).

Phage-mediated horizontal gene transfer (HGT) is recognized as a mechanism for bacterial acquisition of antibiotic resistance (44). Multiple studies have indicated that phages containing resistance genes could enhance antibiotic resistance in bacterial hosts (45, 46). However, direct evidence of phage-mediated HGT leading to antibiotic resistance in mycobacteria is currently insufficient. Experimental data suggest that phages seldom carry functional antibiotic resistance genes (ARGs), and numerous anticipated ARGs do not provide resistance when tested experimentally (47).

Although the proteins encoded by the M-cluster and C-cluster phages(Fig. 3e; Fig. S4c) exhibit structural similarity to aminoglycoside nucleotidyltransferases, their specific contribution to antibiotic resistance is uncertain and necessitates additional experimental validation.

### Unique structures and potential functions of uncharacterized mycobacteriophage proteins

**Fig 4:**
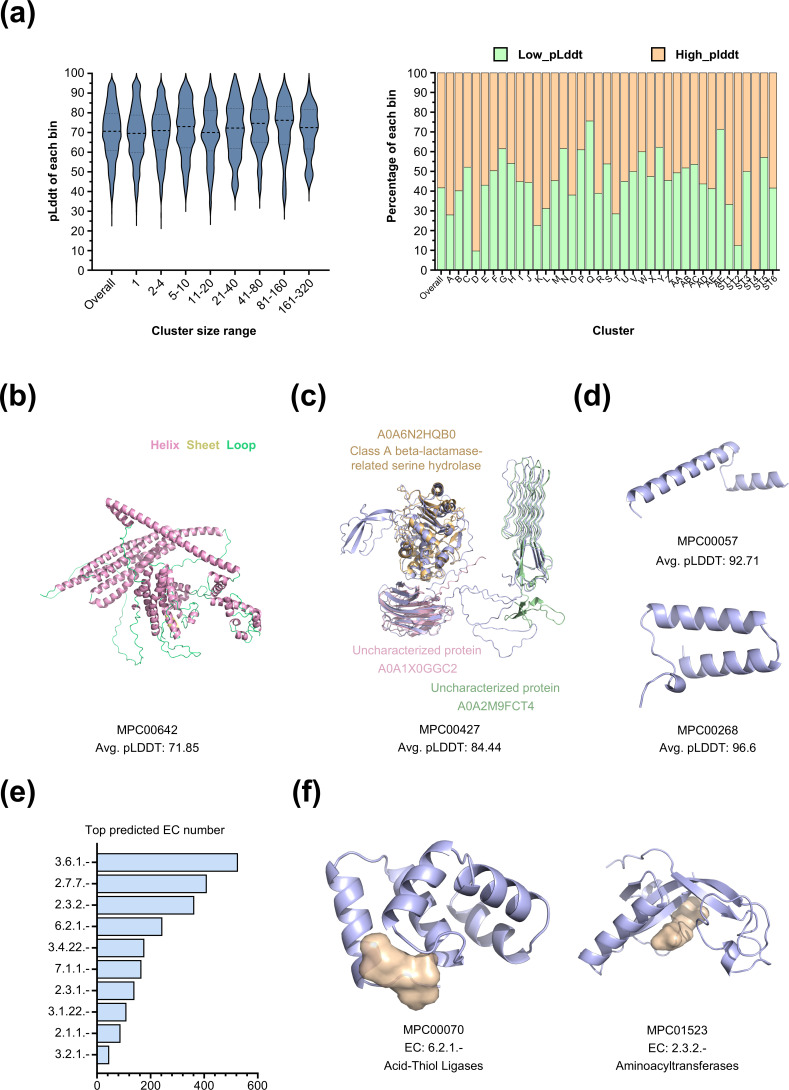
Analysis of structure without homologs uncovers novel enzymes and folds. (**a**) Summary of average reference structure pLDDT distributions for different dark cluster sizes (left): from left to right, each bin’s median pLDDT is 70.61, 69.54, 70.96, 73.01, 70.02, 72.24, 74.63, 76.18, 72.46. Summary of the percentages of High_pLDDT and Low_pLDDT proteins in each cluster (right). Proteins with an average pLDDT score ≥70 were defined as High_pLDDT, while those with a score <70 were defined as Low_pLDDT. (**b**) The AlphaFold model depicts the near-full helix structure encoded by the mycobacteriophage, which lacks a global match in PDB, AFDB_SwissProt, and AFDB. (**c**) The AlphaFold model of the three-domain protein encoded by the mycobacteriophage does not yield global matches in PDB, AFDB_SwissProt, and AFDB. Nevertheless, its three structural domains exhibit near-exact matches. (**d**) The AlphaFold model of the helix-turn-helix structure encoded by the mycobacterium phage aligns with entries in databases associated with diverse biological processes, such as anti-CRISPR, anti-RecBCD, and anti-population sensing. (**e**) Counts of EC number most frequently predicted by DeepFRI on the set of 826 cluster representative structures with an average pLDDT score above 90. (**f**) Two examples of novel enzymes predicted by DeepFRI (MPC00070, MPC01523) exhibit predicted pockets highlighted in light yellow.

The workflow based on structural similarity facilitated the identification of homologous proteins for most mycobacteriophage proteins. However, homologs were not identified for 11.58% of these proteins. Additionally, 16.12% of the proteins had annotations that were considered invalid. To explore potentially unique protein structures encoded by mycobacteriophages, we examined proteins without identified homologs. Among these, 55.19% exhibited an average pLDDT score above 70 (Fig. 4a), suggesting that the inability to identify homologs was not solely due to poor modeling quality. To understand the possible biological processes involving these proteins, we utilized DeepFRI for functional predictions. For proteins where DeepFRI did not provide GO or EC annotations, we conducted further analysis using Foldseek in local comparison mode against the CATH domain classification database (48) (see Materials and Methods). The findings showed that only 8.15% of the query structures (6.93% of the query sequences) had matches in the CATH database (Table S2), suggesting that mycobacteriophages might encode protein structures with unknown functions.

The recently released Big Fantastic Virus Database (BFVD) (49), comprising 351,242 viral protein structures, significantly expands the coverage of the viral proteome in structure prediction-based databases. To further assess the novelty of our predicted protein structures, we compared 14,622 protein structures from mycoPHG_clu_model_db with those in the BFVD using the same parameters for structural similarity searches. Our analysis indicated that 5,603 structures (38.32% of the total) had no detectable matches in the BFVD, suggesting they may represent novel structural configurations within this study’s scope. The remaining 9,019 structures (61.68% of the total) had matches in the BFVD (Table S11), including 5,944 non-singleton and 3,075 singleton structures. These findings suggest that the structural predictions in this study complement the BFVD of phage protein structures, providing a potentially valuable resource for researchers studying mycobacteriophages.

MPC00642 demonstrated a distinctive structure encoded by mycobacteriophages, with an average pLDDT value of 71.85. The conformation is predominantly helical, with connecting loops, comprising 857 residues. This structure did not match any entries in structural databases using a Foldseek search (Fig. 4b). Additionally, MPC00427 was also predicted to lack structural matches in the database. It can be divided into three domains, each aligning with the best AFDB50 match for MPC00427 (Fig. 4c). This indicates a potential gene fusion event in a phage, involving a Class A beta-lactamase-related serine hydrolase domain and two domains of unknown function. Similarly, mycobacteriophages seem to encode numerous helix-turn-helix-like structures (Fig. 4d). These structures are linked to various activities, such as anti-CRISPR (50), anti-quorum sensing (51), and anti-RecBCD functions (52) (Fig. S5). However, determining the precise functions of these relatively simple structures solely based on structural resemblances remains challenging.

Figure 4e displays the most frequently predicted EC numbers among the unannotated phage proteins, with the most enriched EC number being 3.6.1, “Action on Phosphorus-Containing Anhydrides” (526 sequences). Figure 4f illustrates two novel enzyme structures and their active sites, as predicted by DeepFRI. MPC00070 had the highest confidence-predicted EC number of 6.2.1.-, suggesting that it functions as an acid–alcohol ligase (esterase), an enzyme involved in various acid-CoA linkage reactions in microbial energy metabolism. Similarly, MPC01523 exhibited the highest confidence-predicted EC number of 2.3.2.-, indicating its potential role as an aminoacyltransferase that catalyzes diverse aminoacyl transfer reactions as well as the transfer of E3 ubiquitin (53).

### Coevolutionary counter measures between mycobacteriophage and its host bacteria

Throughout the extended coevolutionary interaction between mycobacteriophages and *Mycobacteria*, both have evolved various defense and counter-defense mechanisms. Although many defense systems in mycobacteria are still not fully understood (54), a database of counter-defense protein structures was established to identify the primary anti-phage strategies of mycobacteria from a different perspective.

**Fig 5:**
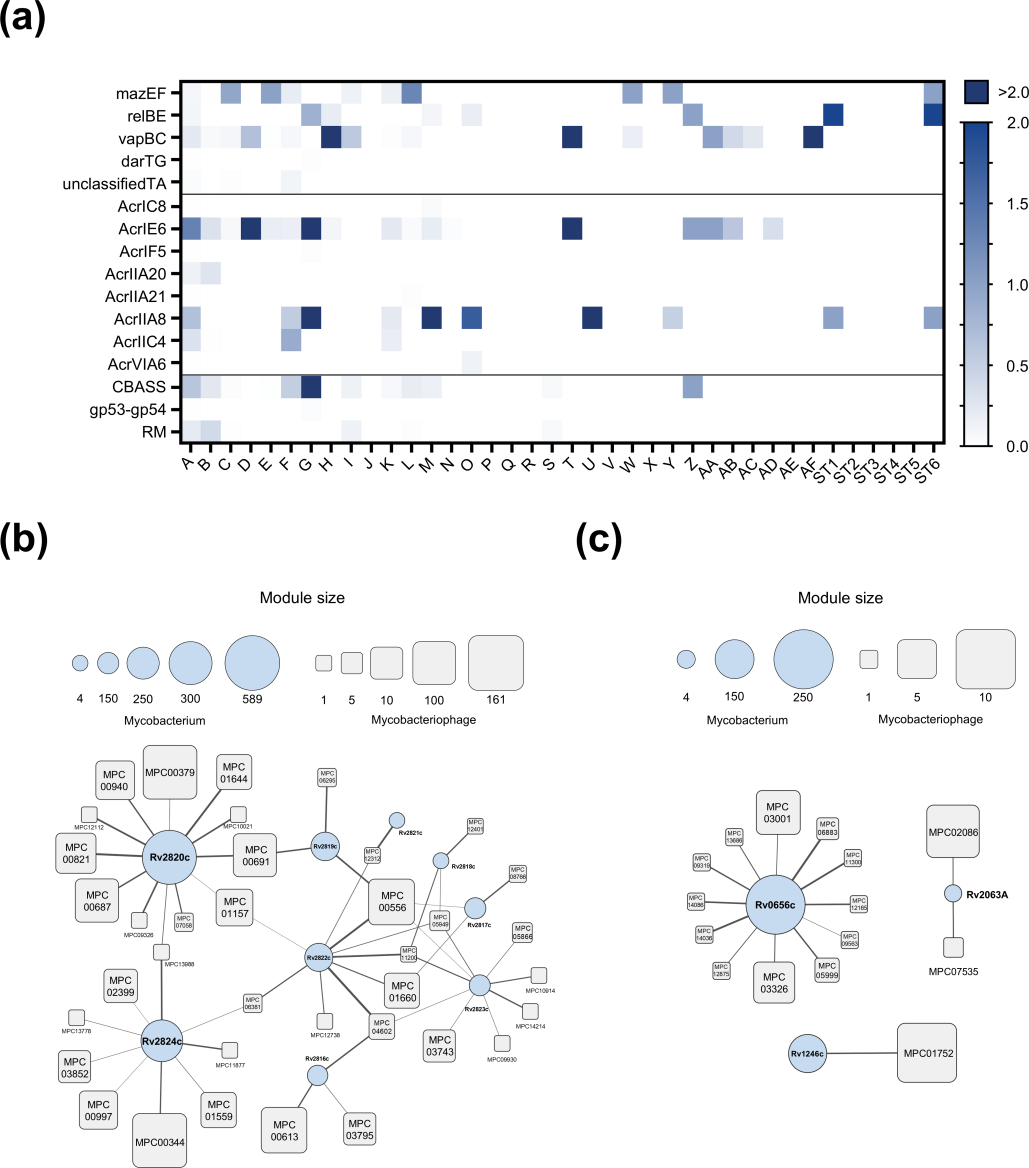
Structural annotation revealing mycobacteriophage-encoded anti-defense arsenal. (**a**) The distribution of anti-defense proteins in mycobacteriophage was determined through structural homology analysis comparing the protein structures of mycobacteriophages with the anti-defense protein structure library. The abundance of these proteins is normalized per phage cluster. (**b**) The interaction network between the anti-CRISPR protein of mycobacteriophage and the CRISPR system-associated protein of *Mycobacterium tuberculosis*. (**c**) The interaction network between the antitoxin protein of mycobacteriophage and the toxin protein of *Mycobacterium tuberculosis* is illustrated. Representative protein structures of mycobacteriophage are shown using rounded rectangles, scaled according to the number of proteins they represent. Mycobacterial proteins are depicted as circles, scaled based on the number of mycobacteriophage proteins they interact with. Edge thickness reflecting interactions scores between mycobacterial protein and mycobacteriophage protein.

The counter-defense protein structure database consists of three sections: a database of Msm/Mtb antitoxin proteins containing 125 proteins (55), a database of anti-CRISPR (Acr) proteins containing 99 proteins (50), and a database of proteins countering other defense systems containing 35 proteins (56) (see Materials and Methods). Foldseek software was utilized to explore the counter-defense protein structure database (see Materials and Methods) and generate heatmaps of anti-defense-related proteins identified in each cluster (Fig. 5a, Tables S12 and 13). The heat levels were standardized according to the number of phages within each cluster (see Materials and Methods). As shown in Fig. 5a, Acr-like proteins and antitoxin proteins were the most abundant among mycobacteriophages.

Since AlphaFold-Multimer (57) demonstrated a strong ability to predict pathogen-host cross-kingdom interactions (58), protein–protein interaction predictions were performed for TA system antitoxin proteins and Acr proteins with their respective host proteins (see Materials and Methods). The interaction network between phage Acr proteins and *M. tuberculosis* CRISPR-related genes indicated that mycobacteriophage Acr-like protein-binding targets were significantly enriched for crRNA processing (Fig. 5b; Table S3), potentially a key defense strategy against CRISPR systems. The top-scored predicted complexes of *Mycobacterium* phage proteins and *M. tuberculosis* CRISPR system crRNA-related proteins are shown in Fig. S6a. The interaction network of mycobacteriophage antitoxins with the corresponding toxins of the host’s TA system suggests that *Mycobacterium tuberculosis* vapBC, mazEF, and relBE TA systems likely serve as phage defense mechanisms (Fig. 5c; Fig. S6b and Table S4).

### Mycobacteriophage–host interaction strategies

**Fig 6:**
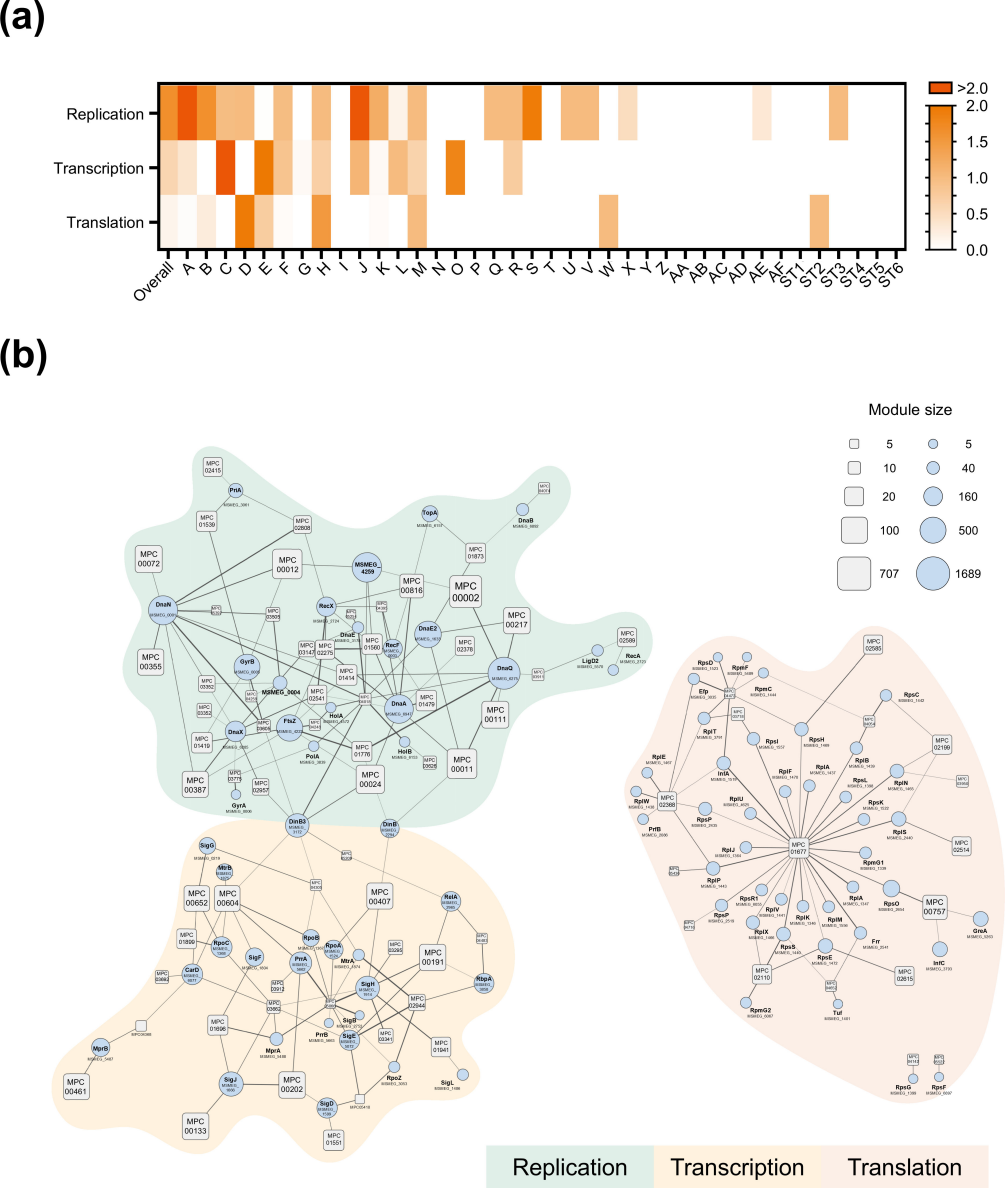
Structural annotation and phage–host interaction prediction revealing phage hijacking strategy of host molecular machines. (**a**) The distribution of replication-related, transcription-related, and translation-related proteins in mycobacteriophage was determined based on structural homology analysis and predicted GO terms by DeepFRI. The abundance of these proteins is normalized per phage cluster. (**b**) The interaction network between the replication-related, transcription-related, and translation-related proteins of mycobacteriophage and the replication-related, transcription-related, and translation-related proteins of *Mycolicibacterium smegmatis*. Representative protein structures of mycobacteriophage were illustrated using rounded rectangles, sized proportionally to the number of proteins they represent. Mycobacterial proteins were depicted as circles, with their size proportional to the number of interacting mycobacteriophage proteins. Edge thickness reflecting interactions scores between mycobacterial protein and mycobacteriophage protein.

Like other phages, mycobacteriophage genomes are highly compact and lack a complete replication, transcription, and translation system (26, 27). Replication-related, transcription-related, and translation-related genes in mycobacteriophages were identified using structural homology searches and GO annotations from DeepFRI. Normalizing the coding frequency of each gene type to the number of phages revealed that replication-related genes were the most commonly encoded, followed by transcription-related genes, as shown in Fig. 6a. Phages in clusters A and J, on average, encoded more than two replication-related genes per strain, while those in cluster C encoded over two transcription-related genes per strain. These results suggest variations in the strategies employed by phages in different clusters to achieve efficient infestation.

To elucidate the strategy of mycobacteriophages for achieving efficient infection, we conducted large-scale protein–protein interaction predictions between these phage genes and mycobacterial replication-related, transcription-related, and translation-related proteins. The phage–host interaction network for these processes was established by screening protein pairs with ipTM + pTM scores exceeding 0.5, as shown in Fig. 6b (Table S5). To further understand how mycobacteriophages exploit the host’s protein machinery for efficient infection, we applied a more stringent threshold to identify the Alphafold-Multimer-predicted complex (ipTM + pTM > 0.7; Table S6). The GO annotations of the host proteins were retrieved from UniProt (Table S7). The top three predicted complexes involving replication-related, transcription-related, and translation-related proteins are shown in Fig. S7a through c.

In the context of DNA replication, our analysis revealed that the host biological process with the highest number of interactions was DNA repair (GO:0006281), involving four proteins: DnaQ, RecX, RecF, and DinB3. RecF notably participates in DNA mismatch repair in *Escherichia coli* phages (59). The second most interactive biological process was DNA replication (GO:0006260), involving five proteins: DnaN, DnaX, DnaA, DnaE, and HolB. DnaN acts as a clamp to tether DNA polymerase to DNA, thereby stimulating DNA synthesis. Additionally, DnaN plays a crucial role in DNA repair after UV-induced damage (60).

For the transcription of mRNAs using DNA as a template, the biological process in the host with the most interactions was DNA-templated transcription initiation (GO:0006352). Apart from RpoC, the host proteins involved in this process are sigma factors (SigH, SigJ, SigE, and SigB). RpoC has been associated with transcriptional resistance to termination in *E. coli* phages, a mechanism used to regulate downstream gene expression in the phage (61). In *Staphylococcus aureus*, SigH has been found to stabilize phage lysogeny. Deleting SigH results in reduced levels of phage integrase mRNA, leading to an increased rate of prophage excision from the genome (62). The biological process with the second-highest number of interacting pairs was the regulation of DNA-templated transcription (GO:0006355). Interestingly, the host proteins involved in this process belong to a two-component system, specifically MprA, MtrA, and PrrA. These three two-component systems in *Mycobacterium tuberculosis* are known to participate in environmental signaling crucial for bacterial survival (63, 64).

During the translation process, protein metabolism (GO:0019538) exhibited the highest number of interactions among host biological processes. Host proteins linked to this process comprise ribosomal components such as RpsO, RpsS, RplA, RplD, RplP, RplS, RplU, RplE, and RpsC. However, the mechanisms by which phages utilize host proteins for translation remain unclear. However, our translation-associated protein interaction network provided insights into the relationship between phages and host translation-associated proteins.

## DISCUSSION

Phage therapy has gained renewed attention owing to increasing bacterial antibiotic resistance (1). However, viral sequences typically show a low affinity for sequences from other species and are relatively understudied (9). As a result, annotations based solely on sequence homology identify homologs with known functions for only about 35% of proteins, significantly impeding our understanding of phage gene functions (19). Structural homology searches can tackle the challenge of identifying distant homologs (11). Nevertheless, the current AlphaFold protein structure database, which is prediction-based, does not encompass phages. Modeling large data sets using AlphaFold poses challenges owing to its substantial computational cost. To address this issue, the SCSH strategy was developed to model large data sets by effectively reducing the modeling size. The recent success of AFDB50 with Alphafold_cluster demonstrates the feasibility of characterizing protein structures within clusters by obtaining representative structures through sequence clustering (30, 31). To ensure that the representative sequences derived from sequence clustering accurately reflect the structural characteristics of the entire cluster, we applied a stringent 90% bidirectional alignment coverage threshold. While this strict parameter yielded clusters with high cluster purity, it may also lead to under-clustering of the data set. This can explain why some singleton proteins were still able to achieve deep MSAs and high-confidence structural models. Despite this trade-off, the strict cluster parameter enhances the reliability of representative structures, which serve as a solid foundation for downstream structural homology-based functional inference. Thus, SCSH-based strategies for virome annotation can reveal the functional linkages between distant homologs and offer valuable insights into their putative functions.

Given that protein structures are evolutionarily 3–10 times more conserved than their sequences, structural similarity searches can be used to identify long-distance homology between phage clusters and between phages and eukaryotes (11). Recent studies have revealed evolutionary connections between the human and prokaryotic antiviral immune systems. However, it remains uncertain whether prokaryotic viruses contain homologs of proteins associated with human immunity and fundamental life processes. Through a large-scale structural homology search, we identified homologs of the human virus-related Ser/Thr protein kinase in mycobacterial phages, which seem to act as intracellular helpers to viruses. Understanding their origin in prokaryotic viruses could illuminate their role in viral assistance. Additionally, a homolog of the NAD^+^ metabolism-related protein, NAMPT, was found in prokaryotic viruses. Notably, the recently discovered Thoeris defense system achieves antiviral immunity by depleting NAD^+^ to produce NAM, thereby causing abortive cellular infections. NAMPT serves as the rate-limiting enzyme for NAD^+^ synthesis from NAM and illustrates intense phage–host competition (38, 39). However, in our structural similarity search, the proportion of viral proteins among the top matches was significantly lower than that of bacterial or eukaryotic proteins. We attribute this to the absence of viral data from the AlphaFold protein structure database. Owing to their diversity, current viral structural resources can only address specific virus categories. The recently launched BFVD is anticipated to address these limitations. Furthermore, mycobacteriophage protein structure data can complement the BFVD and offer a valuable resource for phage research.

The structural homology between temperate and lytic bacteriophages was examined to identify core functions and biological processes essential for sustaining fundamental life activities in phages. Despite ANI ranging from 53% to 59% between temperate and lytic phages, clustering lytic phage genes based on protein sequences for analysis is challenging. However, utilizing structure-based clustering techniques offers a robust solution due to their superior ability to identify distantly related homologs (11). Structure-based clustering revealed that mycobacteriophages encoding glycosyltransferases, which aid in evading host antibody recognition, clustered together. This clustering highlights the semantic consistency of our modeling and structural clustering approaches. Additionally, a mycobacteriophage-encoded aminoglycoside nucleotidyltransferase-like protein responsible for modifying nucleotides of streptomycin and spectinomycin has been identified (43, 45, 46). While phage-encoded ARGs are rare, it has been reported that predicted ARGs often lack functional activity in laboratory tests (47). Nonetheless, their identification remains crucial. Phages are recognized as critical vectors for HGT, highlighting the importance of assessing such genes for the safety of phage therapy, even when their activity is uncertain.

However, the mechanisms by which phages efficiently exploit the host molecular machinery for replication remain poorly understood. To further elucidate the intricate interactions of phages within the host, a series of phage components interacting with mycobacterial replication-associated, transcription-associated, and translation-associated proteins were identified through large-scale protein–protein interaction predictions using AlphaFold-Multimer. These findings revealed that phage-encoded replication-associated proteins predominantly interact with host DNA repair-related proteins, whereas transcription-associated proteins commonly interact with host transcription initiation proteins. Translation-associated phage proteins interact primarily with ribosomal proteins and sigma factors. These insights may illuminate the strategies employed by phages to harness host proteins for replication. Moreover, long-term coevolution between phages and their hosts has led to the evolution of diverse defense and counter-defense mechanisms, which are crucial for the precise engineering of therapeutic phages (54). Our predictions identified a variety of proteins that interact with *Mycobacterium tuberculosis* CRISPR and TA systems; notably, Acr proteins primarily target crRNA processing. These findings offer valuable insights into the evolutionary conflict between phages and *Mycobacterium tuberculosis*, providing a foundation for developing therapeutic phages.

Despite the implementation of the SCSH strategy, approximately 10% of proteins in mycobacteriophages lack identifiable homologs, and an additional 8.9% of proteins have database matches that remain unannotated. The challenge in finding homologs for phage proteins in databases can be attributed to various factors. Poor modeling quality might impede homolog identification, as some proteins necessitate specific folding conditions that AlphaFold2 cannot accommodate (29). Alternatively, these structures could be novel. The distinct structures encoded by phages, along with their potential roles in biological processes and enzymatic functions, were predicted to facilitate the functional analysis of these novel proteins. For instance, a protein in a mycobacteriophage, primarily composed of alpha helices with a pLDDT score of 71.85, was identified. Although many alpha-helix-dominated proteins are transmembrane proteins, which generally exhibit lower pLDDT scores, the GO and enzyme function annotations for this protein were inconclusive. This lack of clear annotation makes it challenging to infer its functions. Furthermore, several structural domain fusion events were observed in the mycobacterial phages. Proteins unmatched in the global search were identified through partial searches that matched their internal structural domains. This phenomenon may be linked to the streamlined nature of phage genomes. Our modeling outcomes also unveiled numerous helix-turn-helix proteins encoded by phages, a type often associated with counter-defense functions. For example, Shah et al. documented that the Aqs1 protein encoded by phages assists in evading quorum-sensing systems (51). Additionally, Pacumbaba and Center discovered that the T7 phage gp5.9 protein serves a similar function (52). Many anti-CRISPR (Acr) proteins adopt a helix-turn-helix conformation (50). Consequently, annotating the functions of these simple proteins based solely on their structures is challenging, and their specific functions can only be determined experimentally.

Structure-based searching is a powerful approach for identifying remote homology and analyzing structural similarities between phages and other species. Therefore, a parameter requiring 80% alignment overlap was utilized for homolog searching to minimize erroneous annotations resulting from partial structures and incomplete matches. However, this criterion may exclude proteins with insertion/deletion mutations from the structural database. Although the percentage of such proteins was particularly low, it still constitutes a limitation of our annotation rate. A comprehensive re-annotation of mycobacteriophages was conducted using an SCSH-based strategy, and many hypothetical proteins that are distant homologs of the currently annotated proteins were identified. The hypothesis that phages may be the evolutionary origin of proteins in other species is supported by our findings, and hypotheses regarding the functions of specific proteins were formulated. This is particularly important for experimental verification of their functions.

## MATERIALS AND METHODS

### Preparing GenBank sequences for mycoPHG_DB

GenBank accession numbers for 2,169 mycobacteriophages were collected from PhageDB (19). These accession numbers were then used to retrieve genome assembly files, which included protein sequences and GFF files, from NCBI DataHub using their corresponding assembly accession numbers to establish mycoPHG_DB. mycoPHG_DB is a sequence database that comprises 240,754 protein sequences extracted from 2,169 mycobacteriophage genomes.

### Sequence clustering and structural homology-based annotation (SCSH) workflow

A three-step approach is employed for the SCSH workflow. First, sequence clustering is performed using the MMseqs2 (28) cluster module for the construction of mycoPHG_clu_DB. The clustering mode is set to Greedy Set cover (--cluster-mode 0), alignment coverage is set to bidirectional (--cov-mode 0), target coverage is set to 0.9 (-c 0.9), minimum sequence identity is set to 0.5 (--min-seq-id 0.5), and sensitivity is set to 7.5 (-s 7.5). Since MMseqs2 cluster uses a cascade clustering process, some members that are close to clustering may no longer meet the criteria, so the clustering sequence reassignment function is enabled (--cluster-reassign). Second, for the construction of mycoPHG_clu_model_DB, the alphaFold (29) is used to model the representative sequences of each cluster of mycoPHG_clu_DB, and PyMOL is employed to export and compute their average pLDDT scores. Third, the structural homology searches are performed using Foldseek (30). The PDB, AFDB_SwissProt, and AFDB databases were downloaded from https://foldseek.steineggerlab.workers.dev on 20 December 2023. The alignment type is set to TMalign (--alignment-type 1), alignment coverage to bidirectional (--cover-mode 0), target coverage to 0.8 (-c 0.8), and the TM-score threshold to 0.5 (--tmscore-threshold 0.5). The prefilter was skipped, and an all-vs-all alignment was conducted (--exhaustive-search).

### Prediction of functions and pockets

The enriched GO/EC terms were predicted by DeepFRI (41), and potential binding pockets were identified by AutoSite (65). The results from DeepFRI were considered only for proteins with an average pLDDT score greater than 90.

### Annotation rate calculation

To calculate the annotation rate from sequence data, the product field was extracted for all sequences in mycoPHG_DB from the corresponding GFF file. Sequences with the term “hypothetical protein” in their product fields were classified as unannotated. The annotation rate based on sequences was determined using the following formula:


$$Sequence\text{-}based\;annotation\;rate=\frac{n_{all}-n_{unannotated}}{n_{all}}$$

Similarly, to compute the annotation rate based on structural data, all protein names in the target structures were evaluated. Protein names that included terms such as putative, hypothetical, uncharacterized, or domain of unknown function were categorized as void matches. The structure-based annotation rate was calculated using the following formula:


$$Structure\text{-}based\;annotation\;rate=\frac{n_{match}-n_{void}}{n_{all}}$$

### Cluster purity analysis

To evaluate the cluster purity of the SCSH-generated sequence clusters, we utilized the pre-downloaded Foldseek AFDB_SwissProt database as a reference set, following the methodology outlined by Barrio-Hernandez et al. in their study (31).

First, the MMseqs2 convert2fasta module was employed to extract sequences.

Second, we proceeded with the sequence clustering step of the SCSH strategy to cluster the sequences of AFDB_SwissProt databases.

Third, the average LDDT and TM scores per cluster were calculated to evaluate structural similarity. Cluster members were manually compiled into distinct databases. The representative structure was then aligned to cluster members using the Foldseek search module with the parameters -e INF --alignment-type 1 --exhaustive-search 1. The alignment of the LDDT and TM scores was reported using the --format-output lddt and alntmscore. The mean score for each cluster was calculated, as illustrated in Fig. 1c.

Fourth, Pfam labels for all cluster members were obtained from UniProtKB to assess Pfam consistency. Only clusters with a minimum of two sequences having Pfam annotations were included. The accuracy of covered Pfam domains was calculated for all Pfam sequence pairs, excluding self-comparisons. True positives were pairs of Pfam domains sharing the same accession number. The consistency scores for each pair were determined by dividing the count of true positives by the number of Pfams in the reference sequence. Subsequently, the average consistency score across all pairs was computed. This methodological approach facilitated evaluating the percentage of sequences in a cluster with matching Pfam annotations.

Finally, the EC number consistency was evaluated for each cluster. EC numbers were retrieved from UniProtKB, and their consistency was evaluated using a methodology analogous to that applied for Pfam consistency. However, the evaluation was conducted separately for each of the four hierarchical levels of EC classification. Only clusters with a minimum of two sequences having EC annotations were included. Sequences with multiple EC numbers were excluded. Annotations without corresponding codes at each hierarchical level were disregarded. Similar to the Pfam consistency analysis, the consistency score for each sequence pair was calculated by dividing the number of true-positive instances by the total number of EC annotations within the pair, excluding self-comparisons. Finally, the mean consistency scores across all sequence pairs were calculated.

To evaluate the cluster purity of the Foldseek structure clusters, cluster members were manually compiled into distinct databases. Subsequently, we aligned the representatives with the cluster members utilizing the Foldseek search module, employing parameters -e INF, --alignment-type 1, and --exhaustive-search 1. The alignment results, specifically the LDDT and TM scores, were reported in the format-output lddt, alntmscore. The mean values for each cluster were computed, as depicted in Fig. S4d.

### Annotation consistency test

We used a two-step approach to evaluate the alignment of structure-based and sequence-based functional annotations.

First, we removed clusters where the representative sequence annotation included ambiguous terms such as hypothetical, membrane, structural, virion, and putative proteins, ensuring that only annotations with functional significance were considered.

Second, we evaluated the semantic consistency between structure-based and sequence-based annotations by determining if the inferred protein functions were biologically equivalent or closely related. Instances of semantically consistent annotations include Phage tail protein ≈ Minor tail protein and DNA polymerase III subunit epsilon ≈ DnaQ-like DNA polymerase III subunit. To measure the percentage of functionally consistent annotations, we computed the ratio of the total number of members across all clusters with functionally consistent annotations to the total number of members across all clusters.

### DeepFRI accuracy test

To evaluate the accuracy of DeepFRI in predicting the EC numbers for the mycobacteriophage data set, a benchmark test set was constructed. For every mycobacteriophage structure used as a query structure, the corresponding target structure in the AFDB and AFDB_SwissProt databases, containing an EC number annotation, was chosen for the benchmark set. DeepFRI was utilized to predict the EC numbers for these mycobacteriophage structures, and the top-scoring predicted EC numbers were compared with the EC numbers annotated in the databases.

The EC number consistency was evaluated using the identical methodology outlined in “Cluster purity analysis.” However, as the final hierarchical level of the top-scoring EC number predicted by DeepFRI was represented as “-” only the initial three hierarchical levels were included in the analysis.

### Last common ancestor analysis

The LCA (last common ancestor) analysis of the best-matching proteins was conducted using the LCA module of MMseqs2 (28). The resulting LCA data were visualized with Pavian (66) to generate Sankey plots.

### Structure-based clustering

Structural clustering was performed using the Foldseek cluster module(30). The alignment type was set to TMalign mode (--alignment-type 1), the coverage mode was configured to cover both query and target (--cov-mode 0), 90% coverage was applied (-c 0.9), and the E-value threshold was set to 0.01 (-e 0.01).

### Local structure comparison

Local structural comparisons were conducted using the Foldseek search module(30). The alignment type was set to TMalign mode (--alignment-type 1), the coverage mode was configured for target coverage (--cov-mode 1), and 90% coverage was applied (-c 0.9). The CATH-Plus 4.3.0 non-redundant S40 pdb datafile was downloaded from http://download.cathdb.info/cath on 20 December 2023(48).

### Build the counter-defense protein structure database

The counter-defense protein structure database (counter-defense_db) comprises three parts. The first part consists of antitoxin genes encoded by Msm/Mtb, obtained from TADB (Table S8). The second part includes 99 Acr proteins labeled as verified, sourced from the CRISPR immunity database (Table S9). The third part comprises 35 anti-defense proteins identified through manually curated text mining (Table S10). For anti-defense proteins included in the PDB database, experimentally resolved structures were used in the anti-defense protein structure database, while AlphaFold was employed for modeling the remaining proteins.

### Counter-defense protein search

The structural homology searches between mycoPHG_clu_model_db and counter-defense_db are performed using Foldseek (30). The alignment type is set to TMalign (--alignment-type 1), alignment coverage to bidirectional (--cover-mode 0), target coverage to 0.8 (-c 0.8), and the TM-score threshold to 0.5 (--tmscore-threshold 0.5). The prefilter was skipped, and an all-vs-all alignment was conducted (--exhaustive-search).

### Heatmap visualization

All heatmaps related to mycobacteriophage clusters were normalized by the ratio of the number of associated proteins to the number of phages within each cluster. The normalized data were subsequently visualized using GraphPad Prism.

### Prediction of protein–protein interactions between phage anti-defense proteins and host defense systems

Phage proteins with a TM score above 0.6, which aligned with entries in the counter-defense_db, were selected (Table S13). These proteins, along with their corresponding defense system-associated proteins, served as inputs for predicting protein–protein interactions.

AlphaFold-Multimer (57) was employed for protein–protein interaction predictions using default parameters and a maximum template date of 2022-12-31 (--max_template_date=2022-12-31).

### Prediction of protein–protein interactions between phage and host transcription–replication–translation-associated proteins

Phage (Table S14) and host (Table S7) proteins linked to replication, transcription, and translation were utilized as inputs for predicting protein–protein interactions.

Alphafold-Multimer (57) was utilized to predict protein–protein interactions with default parameters and a maximum template date of 2022-12-31 (--max_template_date=2022-12-31). Templates were not used for modeling transcription–replication–translation-associated proteins due to prolonged template loading times.

### Interaction network visualization

The interaction network data between mycobacteriophage proteins and host proteins were derived from AlphaFold-Multimer prediction results, with a screening threshold of an interaction score (ipTM + pTM) greater than 0.5. The interaction score (ipTM + pTM) is calculated by AlphaFold-Multimer as 0.8 × ipTM + 0.2 × pTM, following the methodology described in the AlphaFold-Multimer paper (57). The interaction network was visualized using Cytoscape (67) software, with the thickness of the connecting lines proportional to the interaction scores. Representative protein structures of mycobacteriophage were depicted using rounded rectangles, with their size proportional to the number of proteins they represent. Mycobacterial proteins were represented by circles, with the circle size proportional to the number of mycobacteriophage proteins.

## Data Availability

All data used for this study are publicly available in AFDB (https://alphafold.ebi.ac.uk/, v.4, with specific examples corresponding to UniProt IDs Q9P1W9, A0A6N2GQB0, A0AX0GGC2, and A0A2M9FCT4), the CATH database (https://www.cathdb.info/, v.4.3.0), the RCSB PDB (https://www.rcsb.org/, PDB IDs 4NUS, 2I91, 6MDX, and 4O16), the Foldseek database (https://foldseek.steineggerlab.workers.dev/, pre-generated Alphafold/UniProt, Alphafold/Swiss-Prot, and PDB100 databases), and National Center for Biotechnology Information Assembly Database (https://www.ncbi.nlm.nih.gov/assembly/, see Table S1 for the EntrezIDs). All data and metadata generated supporting the pangenome-scale annotation of mycobacteriophages are openly available at https://doi.org/10.5281/zenodo.13220373 (CC-BY 4.0). No original codes have been developed for this article. The sequences clustered by MMseqs2 v.15.6f452 (https://mmseqs.com/), structure search, and cluster analysis was performed using Foldseek v.8.ef4e960 (https://foldseek.com/), DeepFRI v.0.0.1 for GO predictions (https://github.com/flatironinstitute/DeepFRI), and Alphafold v.2.2.0 for monomer and multimer structure prediction (https://github.com/google-deepmind/alphafold/).

## SUPPLEMENTAL MATERIAL

Supplemental figures msystems.00508-25-s0001.pdf Figures S1 to S7.

Supplemental tables msystems.00508-25-s0002.xlsx Tables S1 to S15.

ASM does not own the copyrights to Supplemental Material that may be linked to, or accessed through, an article. The authors have granted ASM a non-exclusive, world-wide license to publish the Supplemental Material files. Please contact the corresponding author directly for reuse.
