# Supplementary material for: Pangenome-scale annotation of mycobacteriophages for dissecting phage–host interactions based on a sequence clustering and structural homology analysis strategy
Source: mSystems. 2025 Jul 29;10(8):e00508-25. doi: 10.1128/msystems.00508-25 (PMC12363180; doi:10.1128/msystems.00508-25)
Supplement: Supplemental figures — Figures S1 to S7. [file msystems.00508-25-s0001.pdf]

Supplemental Figures

Pangenome-scale annotation of mycobacteriophages for dissecting phage–host interactions based on a Sequence Clustering and Structural Homology analysis strategy

Xiao Guo[a], Zheng-Guo He[a,b,#]

[a]College of Life Science and Technology, Guangxi University, Nanning 530004, China.

[b] State Key Laboratory of Virology, Taikang Center for Life and Medical Sciences, TaiKang Medical School (School of Basic Medical Sciences), Wuhan University, Wuhan, 430071, China

#To whom correspondence should be addressed: School of Basic Medical Sciences, Wuhan University, Wuhan, 430071, China.

Email: hezhengguo2024@whu.edu.cn or hezhengguo2019@163.com

Tel: +86-27-68759222, Fax: +86-27-68759222

Key words: *Mycobacterium tuberculosis*; Phage; Structural homology; Sequence clustering

List of supplementary information

Supplemental Figure 1. Annotation summary and reference structure quality assessment

Supplemental Figure 2. Results of cluster purity and annotation consistency analysis

Supplemental Figure 3. Phage proteins superpose with eukaryotic proteins and StpK7

Supplemental Figure 4. Result of structure-based cluster purity test and DeepFRI accuracy test

Supplemental Figure 5. The anti-defense protein with a helix-turn-helix motif

Supplemental Figure 6. Predicted interactions of Anti-CRISPR proteins and Anti-toxin proteins with host-associated proteins

Supplemental Figure 7. Predicted interactions of phage proteins and host-molecule machinery associate protein

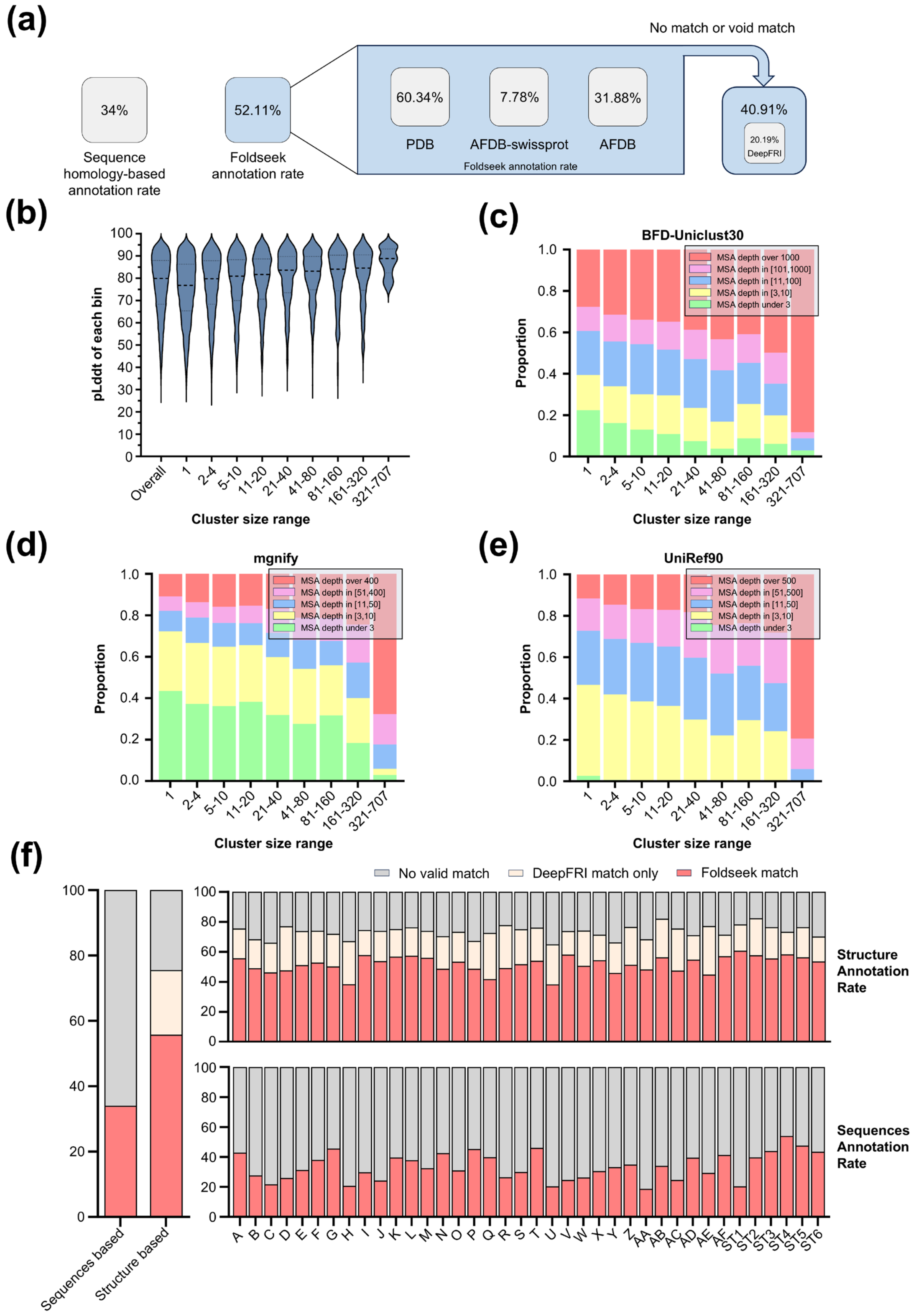

(a)
No match or void match
34%
Sequence homology-based annotation rate
52.11%
Foldseek annotation rate
60.34%
PDB
7.78%
AFDB-swissprot
31.88%
AFDB
Foldseek annotation rate
40.91%
20.19%
DeepFRI
(b)
pLddt of each bin
Cluster size range
Overall
1
2-4
5-10
11-20
21-40
41-80
81-160
161-320
321-707
(c)
BFD-Uniclust30
MSA depth over 1000
MSA depth in [101,1000]
MSA depth in [11,100]
MSA depth in [3,10]
MSA depth under 3
Proportion
Cluster size range
(d)
mgnify
MSA depth over 400
MSA depth in [51,400]
MSA depth in [11,50]
MSA depth in [3,10]
MSA depth under 3
Proportion
Cluster size range
(e)
UniRef90
MSA depth over 500
MSA depth in [51,500]
MSA depth in [11,50]
MSA depth in [3,10]
MSA depth under 3
Proportion
Cluster size range
(f)
No valid match
DeepFRI match only
Foldseek match
Structure Annotation Rate
Sequences Annotation Rate
Sequences based
Structure based

Supplemental Figure 1. Annotation summary and reference structure quality assessment

(a) Annotated contributions based on structural homology searches conducted by PDB, AFDB-swissprot, and AFDB. (b) Summary of reference structure average pLDDT distributions for different cluster sizes, from left to right, each bin's midian pLDDT is 79.92, 76.84, 79.77, 80.94, 81.67, 83.65, 83.20, 84.09, 84.58, 88.86. (c) HHbilts_BFD_uniclust30 MSA depth of clusters binned by their member counter. (d) JackHMMER_mgnify MSA depth of clusters binned by their member counter. (e) JackHMMER_UniRef90 MSA depth of clusters binned by their member counter. (f) Summary of sequence-based annotation rates and structure-based annotation rates (including Foldseek (red) and DeepFRI (yellow)) across different phage clusters is presented in three panels. The left panel illustrates the average sequence annotation rate (34.00%) and the average structural annotation rate (75.55%) for 2,169 mycobacteriophages. The upper right panel displays the average structural-based annotation rate for each phage cluster. The lower right panel displays the average sequences-based annotation rate for each phage cluster.

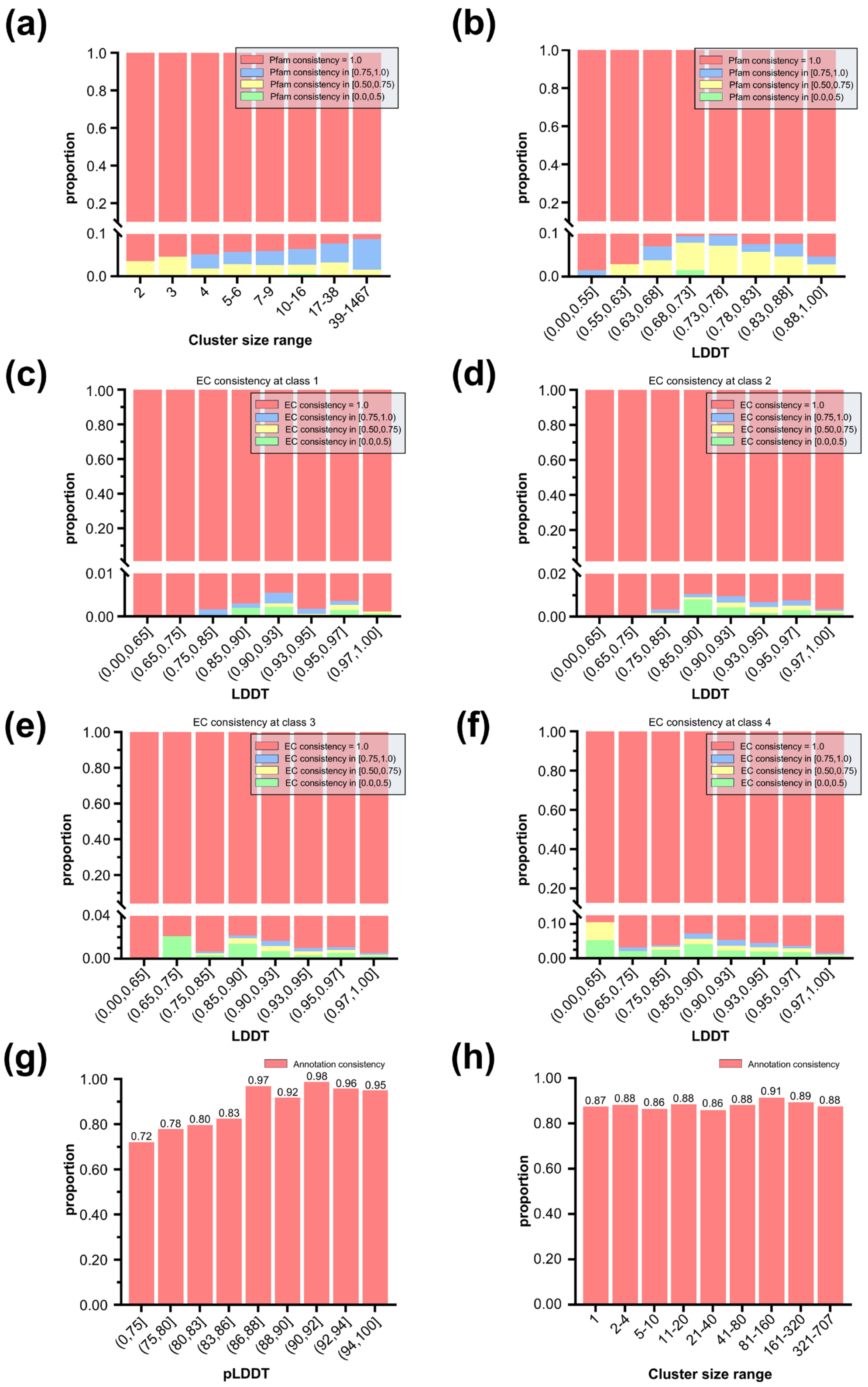
(a)
Pfam consistency = 1.0
Pfam consistency in [0.75,1.0)
Pfam consistency in [0.50,0.75)
Pfam consistency in [0.0,0.5)
proportion
Cluster size range
2
3
4
5-6
7-9
10-16
17-38
39-1467
(b)
LDDT
(0.00,0.55]
(0.55,0.63]
(0.63,0.68]
(0.68,0.73]
(0.73,0.78]
(0.78,0.83]
(0.83,0.88]
(0.88,1.00]
(c)
EC consistency at class 1
EC consistency = 1.0
EC consistency in [0.75,1.0)
EC consistency in [0.50,0.75)
EC consistency in [0.0,0.5)
(0.00,0.65]
(0.65,0.75]
(0.75,0.85]
(0.85,0.90]
(0.90,0.93]
(0.93,0.95]
(0.95,0.97]
(0.97,1.00]
(d)
EC consistency at class 2
(e)
EC consistency at class 3
(f)
EC consistency at class 4
(g)
Annotation consistency
0.72
0.78
0.80
0.83
0.97
0.92
0.98
0.96
0.95
pLDDT
(0,75]
(75,80]
(80,83]
(83,86]
(86,88]
(88,90]
(90,92]
(92,94]
(94,100]
(h)
0.87
0.88
0.86
0.88
0.86
0.88
0.91
0.89
0.88
1
2-4
5-10
11-20
21-40
41-80
81-160
161-320
321-707
Cluster size range

Supplemental Figure 2. Results of cluster purity and annotation consistency analysis

(a) Relationship of mean pairwise Pfam consistency to cluster size range. (b) Relationship of mean pairwise Pfam consistency to LDDT of each cluster. (c) Relationship of mean pairwise EC number consistency at class 1 to LDDT of cluster. (d) Relationship of mean pairwise EC number consistency at class 2 to LDDT of cluster. (e) Relationship of mean pairwise EC number consistency at class 3 to LDDT of cluster. (f) Relationship of mean pairwise EC number consistency at class 4 to LDDT of cluster. (g) Relationship of annotation consistency to mean pLDDT of cluster reference cluster. (h) Relationship of annotation consistency to cluster size range.

Fig S2a-b are plotted with 36,251 clusters with at least two pfam annotated sequences.

Fig S2c-f are plotted with 17,579 clusters with at least two Enzyme Commission number annotated sequences.

Fig S2g-h are plotted with 1,768 clusters with cluster correspondence reference sequence annotation excluding hypothetical protein, membrane protein, structural protein, virion protein, and putative.

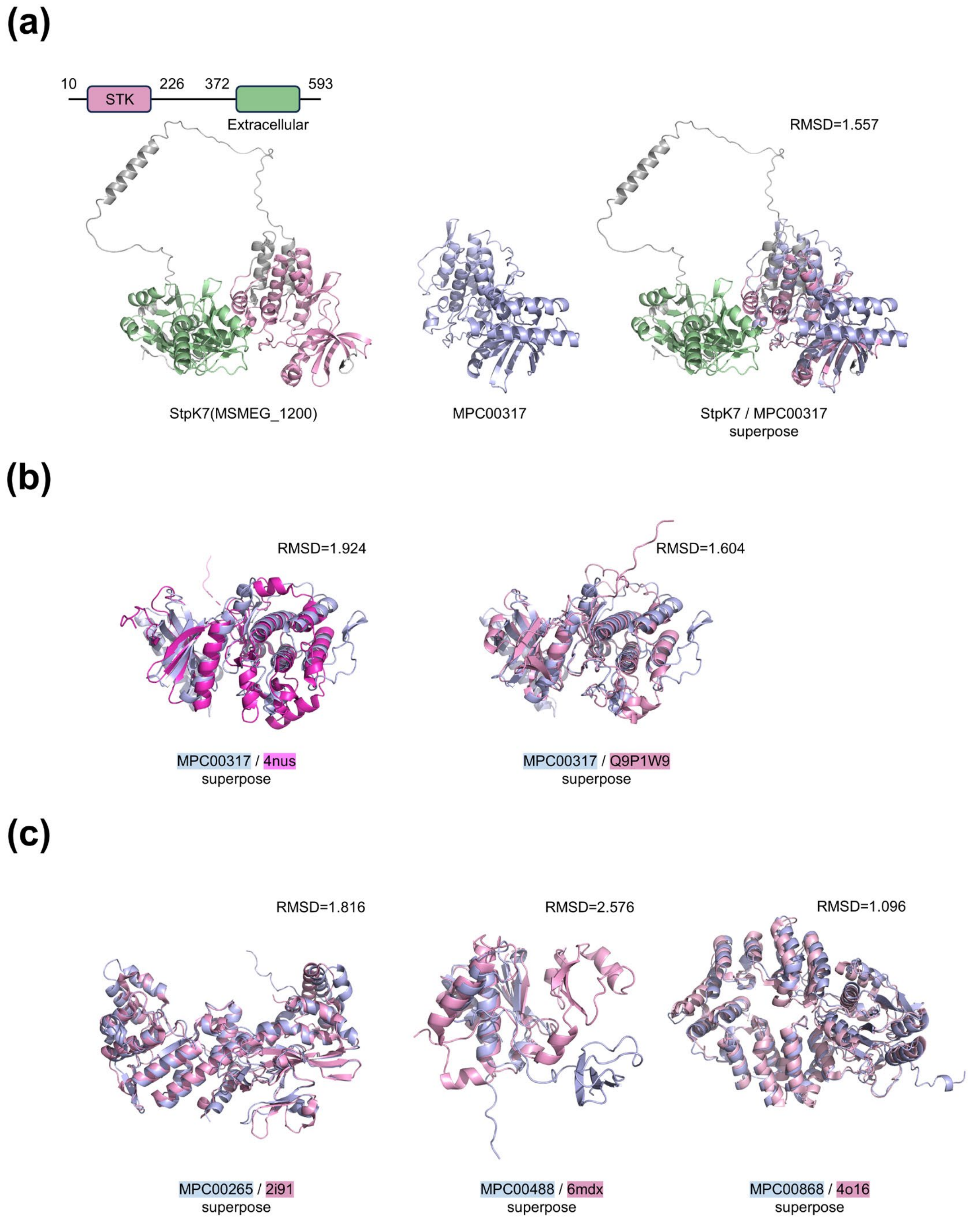

Supplemental Figure 3. Phage proteins superpose with eukaryotic proteins and StpK7

(a) Predicted structure of the eukaryotic-like protein StpK7 encoded by *M. smegmatis*, which is colored according to the structural domains, and its superpose to the structure of MPC00317 with an RMSD of 1.557. (b) The structural superpose correspond to Figure2b. MPC00317 with 4nus result an RMSD of 1.924 (Left), and MPC00317 with Q9P1W9 result an RMSD of 1.604(right). (c) The structural superpose

correspond to Figure2c. MPC00265 with 2i91 result an RMSD of 1.816 (Left), MPC00488 with 6mdx result an RMSD of 2.576 (Middle) and MPC00868 with 4o16 result an RMSD of 1.096 (Right).

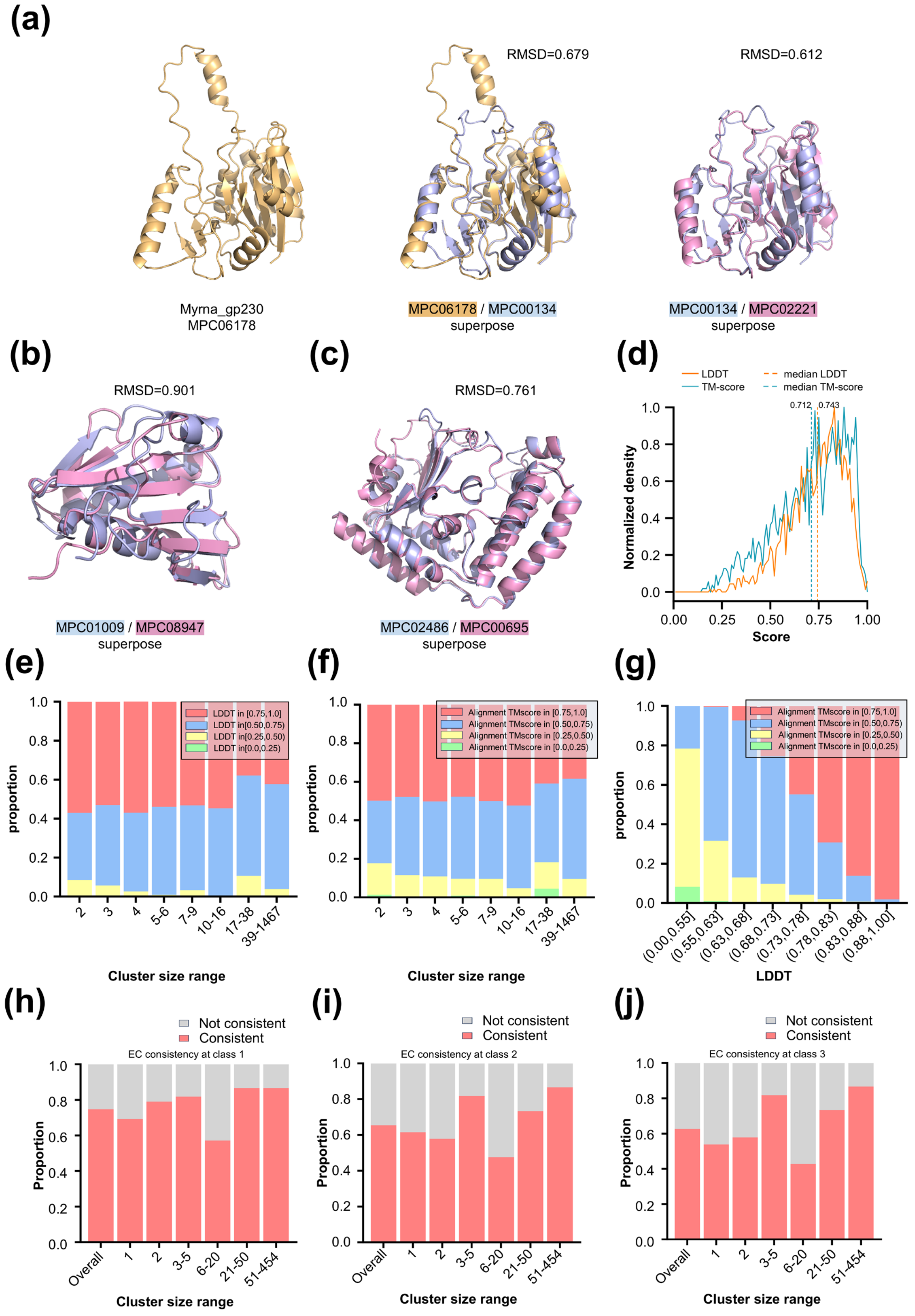
(a)
RMSD=0.679
RMSD=0.612
Myrna_gp230
MPC06178
MPC06178 / MPC00134
superpose
MPC00134 / MPC02221
superpose
(b)
(c)
(d)
RMSD=0.901
RMSD=0.761
MPC01009 / MPC08947
superpose
MPC02486 / MPC00695
superpose
LDDT
TM-score
median LDDT
median TM-score
Normalized density
Score
(e)
(f)
(g)
proportion
Cluster size range
LDDT
(h)
(i)
(j)
Not consistent
Consistent
EC consistency at class 1
EC consistency at class 2
EC consistency at class 3
Proportion
Cluster size range

Supplemental Figure 4. Result of structure-based cluster purity test and DeepFRI accuracy test

(a) Recently reported structures of phage proteins associated with glycosylation of phage coat proteins and their structural superpose. Structure of the cluster C phage Myrna glycosylation-associated protein gp230 (Left), and its structural superposition with the glycosylated protein encoded by cluster F and I temperate phage result an RMSD of 0.679 (Middle). And the structural superpose correspond to left figure of Figure2c, MPC00134 with MPC02221 result an RMSD of 0.612 (Middle). (b) The structural superpose correspond to middle figure of Figure2c, MPC01009 with MPC08947 result an RMSD of 0.901. (c) The structural superpose correspond to Figure2d, MPC02486 with MPC00695 result an RMSD of 0.761 (Right). (d) mycoPHG_clu_model_DB clusters structural consistency. Our clusters have a median LDDT of 0.743 and a median TM score of 0.712 across all clusters. (e) Relationship of pairwise LDDT to cluster size range. (f) Relationship of pairwise TM-score to cluster size range. (e) Relationship of pairwise TM-score to LDDT. (h) Relationship of DeepFRI EC number prediction accuracy at class 1 to cluster size range. (i) Relationship of DeepFRI EC number prediction accuracy at class 2 to cluster size range. (j) Relationship of DeepFRI EC number prediction accuracy at class 3 to cluster size range.

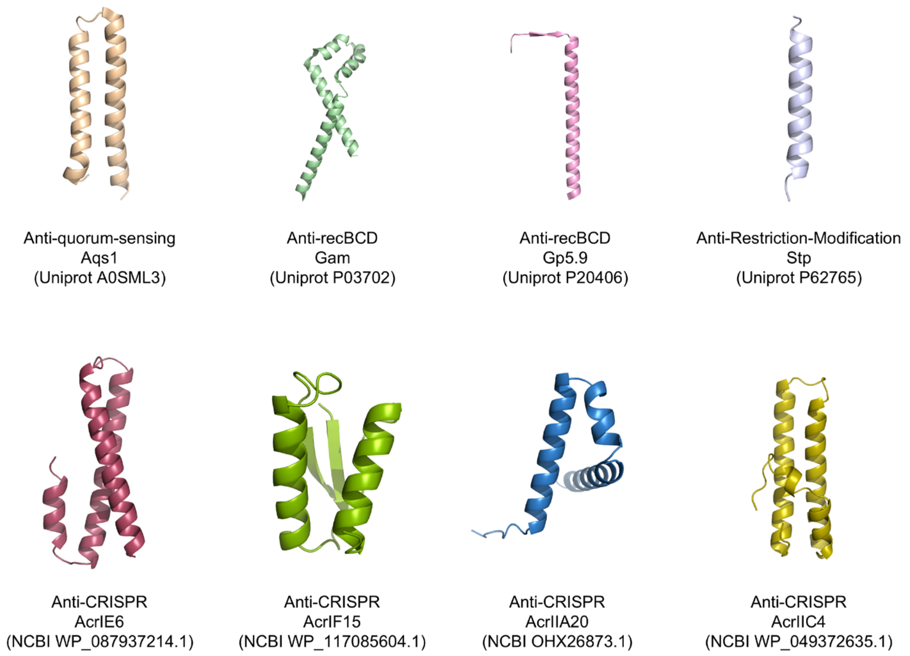

Supplemental Figure 5. The anti-defense protein with a helix-turn-helix motif
Some anti-defense proteins with HTH structural domains obtained by literature mining, included Anti-quorum-sensing, anti-recBCD, anti-restriction-modification and anti-CRISPR.

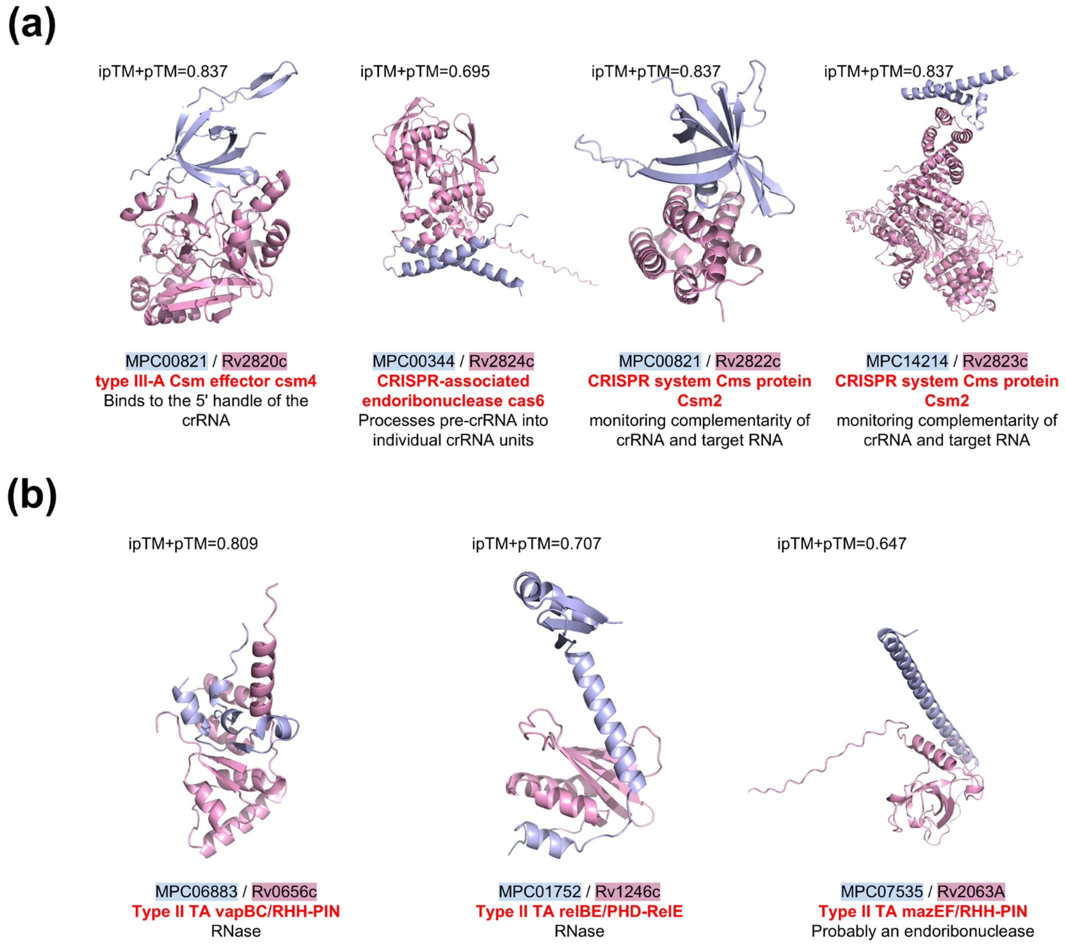

Supplemental Figure 6. Predicted interactions of Anti-CRISPR proteins and Anti-toxin proteins with host-associated proteins

(a) Mycobacterium phage proteins that interact with the *M. tuberculosis* CRISPR system crRNA-processing associated proteins. CRISPR-related proteins annotations are shown in bold red text, and their functions are shown in black text. (b) Mycobacterium phage proteins that interact with *M. tuberculosis* Toxin-Antitoxin system toxin proteins. Category of TA system are shown in bold red text, and their toxin functions are shown in black text.

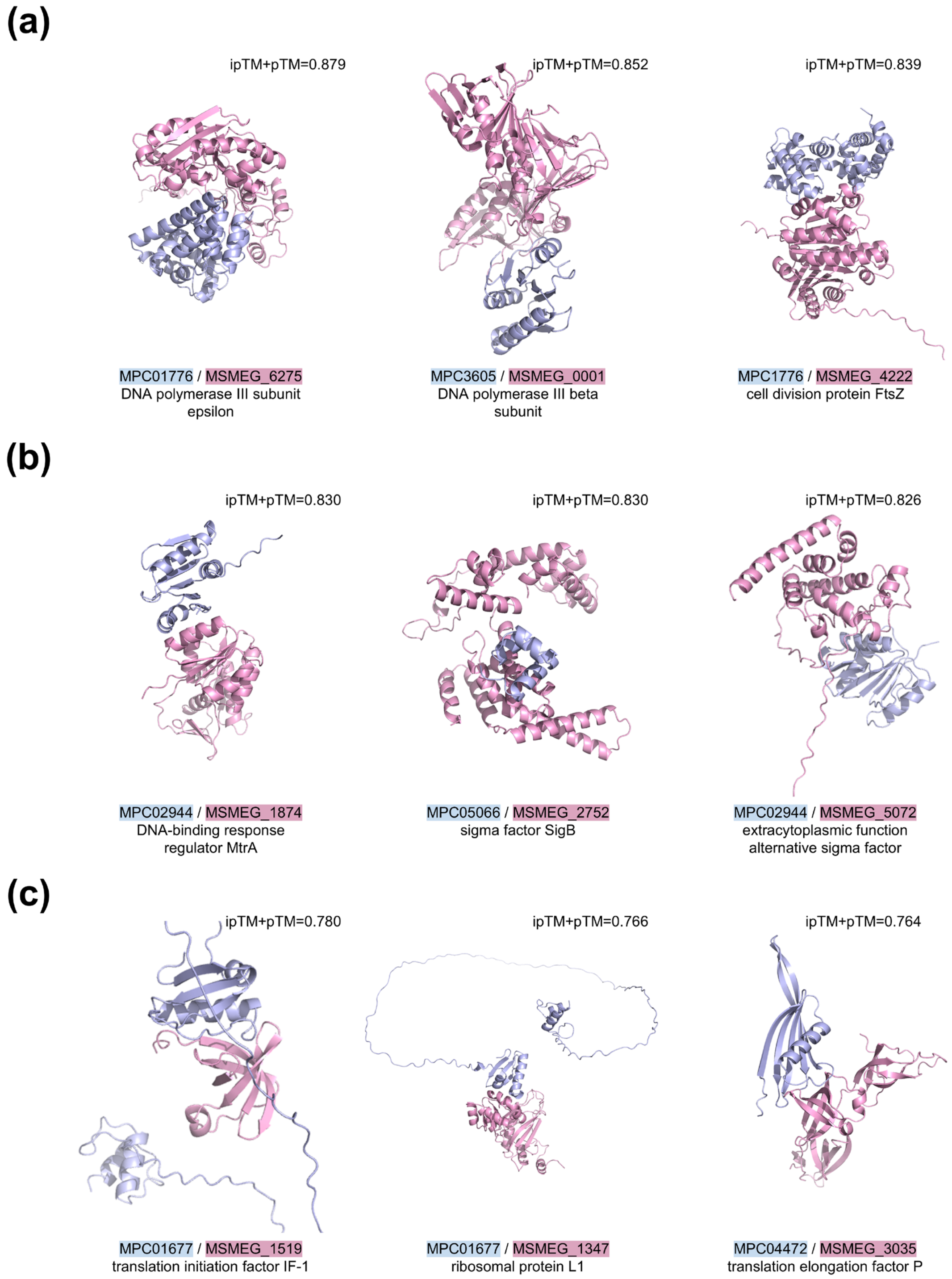

Supplemental Figure 7. Predicted interactions of phage proteins and host-molecule machinery associate protein

(a) The top three phage replication-related proteins are predicted to interact with *M. smegmatis* replication-related proteins. (b) The top three phage transcription-related proteins are predicted to interact with *M. smegmatis* transcription-related proteins. (c) The top three phage translation-related proteins are predicted to interact with *M.*

*smegmati*s translation -related proteins. Phage proteins are shown in blue, mycobacterial proteins are in pink, and functional annotations of mycobacterial proteins are indicated in black across all panels.
